# N-glycosylation-mediated CD147 accumulation induces cardiac fibrosis in the diabetic heart through ALK5 activation

**DOI:** 10.7150/ijbs.77469

**Published:** 2023-01-01

**Authors:** Mingchuan Liu, Tingwei Peng, Lang Hu, Min Wang, Dong Guo, Bingchao Qi, Gaotong Ren, Di Wang, Yunqing Li, Liqiang Song, Jianqiang Hu, Yan Li

**Affiliations:** 1 Department of Cardiology, Tangdu Hospital, The Fourth Military Medical University, Xi'an, 710038, China.; 2 Department of Pulmonary and Critical Care Medicine, Xijing Hospital, The Fourth Military Medical University, Xi'an, 710038, China.; 3 Brigade 2, College of Basic Medicine, The Fourth Military Medical University, Xi'an, 710038, China.

**Keywords:** CD147, diabetic cardiomyopathy, cardiac fibrosis, ALK5

## Abstract

Emerging evidence has implicated the important role of fibrosis in diabetic cardiomyopathy (DCM), while the underlying mechanism remains unclear. Considering the distinct and overlapping roles of Cluster of Differentiation 147 (CD147) in the pathogenesis of fibrotic diseases, we aim to investigate the role of CD147 in the fibrosis of DCM and explore its underlying mechanism. AAV9-mediated cardiac-specific CD147 silencing attenuated cardiac fibrosis and cardiac function in diabetic mice. CD147 knockdown significantly inhibited high glucose (HG)-induced activation of CFs. Mechanistically, CD147 directly bound to type I transcription growth factor β (TGF-β) receptor I (ALK5), promoting ALK5 activation and endocytosis to induce SMAD2/3 phosphorylation and nuclear translocation. In addition, HG prevented the ubiquitin-proteasome-dependent degradation of CD147 by promoting GNT-V-mediated N-glycosylation. As a result, cardiac-specific CD147 overexpression in control mice mimicked diabetes-induced cardiac fibrosis, aggravating cardiac function. Importantly, CD147 was also upregulated in serum and myocardial specimens from patients with diabetes compared with non-diabetes, accompanied by echocardiographic indices of cardiac dysfunction and excessive collagen deposition. Our study provides the first evidence that CD147 acts as a pivotal factor to promote diabetic cardiac fibrosis, and may contribute to the development of future CD147-based therapeutic strategies for DCM.

## Introduction

Diabetes mellitus (DM) is becoming the leading cause of death worldwide due to several accompanying complications including diabetic nephropathy, cardiomyopathy, neuropathy, and retinopathy 1. Diabetic cardiomyopathy (DCM) is characterized by ventricular remodeling and interstitial fibrosis, and ultimately leads to heart failure. It has a high incidence rate 2, 3, but its pathogenesis is still not fully understood. Treatment options are also limited. Cardiac damage in DCM has recently been linked to myocardial fibrosis, which irreversibly alters normal tissue architecture and transforms the heart function from compensated to decompensated. Patients with DM had shorter contrast-enhanced T1 on cardiac magnetic resonance imaging, and the degree of interstitial fibrosis in right ventricular biopsy specimens of these patients negatively correlates with ejection fraction 4, 5. Despite its clinical implications, the pathology of DM-related cardiac fibrosis remains unclear.

Aberrant proliferation and differentiation of cardiac fibroblasts have been considered responsible for cardiac fibrosis in DM. Excessive cytokines in the diabetic heart, especially transforming growth factor β (TGF-β), can activate fibroblasts by inducing their differentiation into myofibroblasts through increasing expression of contractile proteins, including α-smooth muscle actin (α-SMA) and proteins related to the extracellular matrix (ECM) 6-8. In addition, high glucose (HG) can induce the activation and endocytosis of type I and type II TGF-β receptors (activin receptor-like kinase ALK5 and ALK1), which can further enhance the downstream signaling transduction of R-SMADs 8, 9. However, further study is needed to understand in detail how HG promotes TGF-β signaling transduction and the subsequent activation of fibroblasts in DM-associated myocardial fibrosis.

CD147 (Cluster of Differentiation 147) is a member of the immunoglobulin superfamily. This transmembrane glycoprotein is upregulated in many types of cancers 10. Highly glycosylated-CD147 can effectively stimulate the production of matrix metalloproteinases, promoting tumor growth, invasion, and migration, considered potential targets for multiple cancers 10, 11. CD147 is involved in the progression of atherosclerosis, hypertension, and platelet activation 12-14. Importantly, CD147 is up-regulated in the left ventricle (LV) of patients with myocardial infarction, inflammatory cardiomyopathy and dilated cardiomyopathy 15-17. Recent studies indicated that CD147 might be involved in the development and progression of myofibroblast differentiation and fibrosis in different organs such as liver and kidneys 18, 19. However, whether it is involved in the pathological fibrosis of DCM and the underlying mechanism remains unanswered questions.

In this study, we found that CD147 was significantly upregulated in both cardiac tissues of diabetic mice and right atrial tissue samples from patients with diabetes, accompanied by cardiac remodeling and impaired cardiac function. AAV9-mediated cardiac-specific CD147 silencing attenuated cardiac fibrosis and cardiac function in diabetic mice. *In vitro* assays further showed that CD147 knockdown significantly inhibits HG-induced activation and proliferation of cardiac fibroblasts (CFs). CD147 directly binds to type I TGF-β receptor I (ALK5), promoting ALK5 activation and endocytosis to induce SMAD2/3 phosphorylation and nuclear translocation. In addition, the HG microenvironment prevented ubiquitin-proteasome-dependent CD147 degradation by promoting GNT-V-mediated N-glycosylation. Moreover, cardiac-specific CD147 overexpression mimicked diabetes-induced cardiac fibrosis and associated cardiac dysfunction in control mice. Overall, our results suggested that N-glycosylation-mediated CD147 accumulation induces sustained activation of CFs, which may provide a new therapeutic target for cardiac fibrosis in DCM.

## Materials and methods

### Plasma and cardiac tissue samples of patients

Plasma samples (n = 20 per group) and atrial septum samples (n = 3 per group) were collected from patients with DM/impaired fasting glucose (IFG)/impaired glucose tolerance (IGT) and individuals without DM from the Tangdu Hospital of the Fourth Military Medical University. Patients with coronary heart disease, hypertension, or any other metabolic cardiomyopathy were excluded. All patients enrolled in this study provided their signed informed consent. All experiments were approved by the Ethics Committee of the Tangdu Hospital of the Fourth Military Medical University.

### Animal models

C57 male mice (6-8 weeks) were purchased from the Experimental Animal Center of the Fourth Military Medical University. All animal procedures were approved by the Fourth Military Medical University Laboratory Animal Ethics Committee. Diabetes was induced by an intraperitoneal injection of 0.1g/kg streptozotocin (STZ, Sigma Aldrich, St. Louis, MO, USA) for five consecutive days 20. Mice with random blood glucose > 16.6 mmol/L were considered diabetic. Forty-five days after successful model establishment, the mice received an intracardiac injection of adeno-associated virus vector 9 (AAV9)-shCD147 or AAV9-sh GNT-V to knock down CD147 or GNT-V or AAV9-CD147 to overexpress CD147. Then the mice were killed and heart samples were collected at the end of the third month.

### Mass spectrometry-based quantitative proteomic analysis

Mouse heart samples were placed in SDT lysate and transferred to a 2 mL centrifuge tube pre-loaded with an appropriate amount of quartz sand, and homogenized using an MP homogenizer. Then, the supernatant was removed and heating in a boiling water for 15 min under ultrasonication and centrifuged at 14,000 *g* for 15 min. The filtrate was passed through a 0.22 μm filter membrane, and the total amount of protein in the sample was quantified by the bicinchoninic acid assay. The Easy LC system was used for separation. The samples were separated from the analytical column using an automatic sampler at a flow velocity of 300 nL/min. Then, the samples were separated by chromatography and analyzed by Q-Exac-TIVE mass spectrometry. MaxQuant software was used to query Uniprot protein database (website: https:// www.uniprot.org) for protein data analysis. Statistical significance was decided based on fold change > 1.5 and *P* < 0.05.

### Glucose tolerance assay

The mice were intraperitoneally injected with distilled water or 2 g/kg glucose after being fasted for 12 h. Blood samples were collected from the caudal vein at 0, 5, 15, 30, 60, and 120 min after injection. Then, blood glucose was measured using a Yuwell glucometer (Yuwell, China).

### Recombinant AAV9 production

Recombinant AAV9-shCD147 and AAV9-CD147 were constructed by Gene Chem Technology (Shanghai, China). A plasmid was constructed to generate AAV9-shCD147, AAV9-CD147, AAV9-shNC, and AAV9-EV viral particles via triple transfection of HEK 293T cells. Adeno-associated virus of AAV9-cTNT-m-CD147 and AAV9-cTnT-m-Null were prepared by Hanbio Biotechnology Ltd (Shanghai, China). Expression of mouse CD147 gene is driven by cTnT promoter to ensure cardiomyocyte specific expression. The virus was purified by iodixanol gradient centrifugation, and the viral titer was quantified by qPCR. The mice were anesthetized with 2% isoflurane before the intracardiac injection of 1 × 10^11^ viral particles.

### Echocardiography

The cardiac function of all experimental mice was evaluated by echocardiography using a Vevo 2100 echocardiography system (VisualSonics, Toronto, ON, Canada). Left ventricle internal dimension at end-diastole and end-systole (LVIDd and LVIDs, respectively), left ventricular ejection fraction and shortening fraction (LVEF and LVFS, respectively), mitral valve flow velocity, and the ratio of E wave to A wave (E/A ratio) were calculated. Six consecutive cardiac cycles were sampled, and the average heart rates were found to be around 400 bpm.

### Histological analysis and immunohistochemistry

The myocardial tissue was fixed with 4% paraformaldehyde, embedded in paraffin, and cut into 5 μm slices. Sections were stained with HE and Masson's trichrome staining. Other sections were incubated with mouse anti-collagen I antibody (Cell Signaling Technology, USA, 1: 2000 dilution) or mouse anti-collagen III antibody (Cell Signaling Technology, 1:2000 dilution) diluted in 3% bovine serum albumin in 1xTBS overnight at 4 °C. Next, the sections were incubated with a biotinylated anti-mouse IgG horseradish peroxidase-conjugated secondary antibody at room temperature for 2 h. Myocardial morphology and collagen deposition were observed by observation under a fluorescence microscope (EVOS^TM^ Thermo-fisher, USA). For quantification of each sample, a modified H score ([{% weak staining}{1]+[{% moderate staining}{2]+[{% strong staining}{3]) was used to determine the overall percentage of COL1a, COL3a, CD147 and GNT-V positive in the entire stained heart sample, which ranged from 0 to 300 21.

### Immunofluorescence (IF) staining

IF was used to detect the degree of fibrosis and analyze co-localization. Heart slices or cardiac fibroblasts were fixed with 4% paraformaldehyde for 24 h and then dehydrated with 30% sucrose for 48 h. Non-specific staining was blocked with 10% goat serum. Then, each slice was incubated with the diluted primary antibody overnight at 4 °C. Then, the slices were incubated with the secondary antibody conjugated with Alexa Fluor 488 and Cy3 (1/1000, Beyotime, Shanghai, China) in the dark at room temperature for 2 h. The nuclei were stained with DAPI (Beyotime). After washing thrice with 0.02% PBST for 5 min, the slides were observed under a fluorescence microscope (Fluoview 1000, Olympus).

### Neonatal mice cardiac fibroblast culture

Primary neonatal mice cardiac fibroblasts were isolated from 0-2 days old neonatal mice. The extraction and separation were performed as described previously 22. The cells were then cultured in a medium with low glucose (5.5 mmol/L) and HG (33 mmol/L) in a humidified 5% CO2 incubator at 37 °C for 24 h and then infected with adenoviruses according to specific multiplicity of infection.

### Construction of siRNA against CD147 and GNT-V

The mice CD147 and GNT-V RNAi target sequences are 5'-GAGUGGGUCUGGUUUAAGATT-3' and 5'-GGAATCAAACTTCAAGATTGC-3', respectively. Nonrelated scrambled RNAi, without any matching for the mouse genomic sequence, were used as respective controls (5'UUCUCCGAACGUGUCACGUTT-3' and 5'-GCCGGTTCCTTGTTACACCTT-3'). Adenoviruses harboring these RNAi constructs were generated using the pSilencer™ adeno 1.0-CMV System (Shanghai, China) according to the manufacturer's instructions. Empty adenoviral vectors (Ad-NC) and CD147 or GNT-V-specific small hairpin RNA (Ad-shCD147, Ad-shGNT-V) were constructed by Hanbio Biotechnology Ltd (Shanghai, China). The viral titer was about 1 × 10^11^ PFU/mL.

### Quantitative real-time PCR analysis

Total RNA in cardiac tissues was extracted using TRIzol (TaKaRa, Japan) and then reverse transcribed into cDNA. All primers were purchased from Sangon Biotech. All procedures were conducted using TB Green® Premix Ex Taq (#RR820L, TaKaRa) following the manufacturer's protocols. Quantification was performed by the 2-^ΔΔCT^ method after normalization of the expression levels against β-actin. The primer sequences are listed below:

### Western blot analysis

Cardiac tissues and cultured cardiac fibroblasts were collected, and the total protein was lysed using radio-immunoprecipitation assay buffer, separated by sodium dodecyl sulfate-polyacrylamide gel electrophoresis and then transferred into a polyvinylidene fluoride membrane (Millipore, Beijing, China). The different primary antibodies were applied overnight at 4 °C, and the details are shown in Table S2. Then the membrane was incubated with secondary antibodies. Chemiluminescence reagents (Millipore, Beijing, China) were used for detection, and the membrane was imaged with a ChemiDoc MP Imaging System (Bio-Rad, USA).

### CCK8 assay

A CCK8 assay (Beyotime) kit was used to quantify the proliferation of cardiac fibroblasts. CFs (2×10^4^ cells/mL) were seeded into a 96-well plate with each well containing 100 µL DMEM with 10% FBS at 37 °C. For treatment, the medium in each well was then replaced with 10 μL CCK8 and 90 μL FBS-free DMEM. After incubation at 37 °C for 2 h. The absorbance values at 450 nm were measured using an Epoch microplate reader at 0, 12, 24, and 48 h.

### EdU (5-Ethynyl-2′-deoxyuridine) staining

After treatment, cardiac fibroblasts were fixed with 4% paraformaldehyde for 20 min at 4 °C. EdU (Beyotime) was then added and incubated for 2 h. After washing with PBS, cells were incubated with fixative solution, glycine (2 mg /mL) and EdU osmotic solution successively. The cells were then subjected to Apollo staining in the dark at room temperature for 30 min. The nuclei were stained with DAPI for another 30 min. The green fluorescence by EdU and blue by DAPI were observed under a fluorescence microscope (EVOS^TM^ Thermo-fisher, USA). Image J 1.50 was used for quantitative analysis.

### Flow cytometry

The apoptosis rate was detected by flow cytometry using Annexin V-APC staining kit (Ebioscience, China). Cardiac fibroblasts were digested with trypsin (Gibco, USA), centrifuged in a microcentrifuge at 1600 rpm for 5 min, washed at 4 °C with D-Hanks buffer (pH = 7.2-7.4), resuspended in 400 μL 1× binding buffer, and stained with 5 μL Annexin V-APC. The cells were then incubated in the dark at room temperature for 15 min. After incubation, 0.5 mL 1 × binding buffer was added and mixed. After centrifugation, 100 μL 1 × binding buffer was added to suspend the cells. Then, 5 μL 7-AAD was added, and the cells were incubated in the dark at 4 °C for 5 min. At the end of incubation, 0.5 mL PBS was added to each sample tube to suspend the cells, and flow cytometry analysis was performed within 4 h.

### LC-MS/MS analysis of CD147-interacting proteins

Liquid chromatography tandem mass spectrometry (LC-MS/MS) analysis was performed using the Fast Silver Stain Kit (Beyotime) following the manufacturer's protocols at Novogene Genetics (Beijing, China) as previously described 23. The 50 most abundant precursor ions were selected for analysis from the full MS scan at a resolution of 15,000 (at 200 m/z). The original documentation for mass spectrometry tests was authenticated by MaxQuant (www.maxquant.org). Spectral data were retrieved from the UniProt Mouse database (www.uniprot.org).

### Immunoprecipitation (IP) and co-immunoprecipitation (Co-IP) assays

IP and Co-IP assays were performed using a Pierce Classic Magnetic IP/Co-IP Kit (Thermo Fisher Scientific) following the protocol provided by the manufacturer. In brief, the IP buffer was used to lyse cultured cardiac fibroblasts. Then 10 μg antibody was added to bind protein overnight at 4 °C. The next day, protein A/G beads were used to conjugate with the antibody at room temperature for 3 h. After washing with IP buffer solution for 5 times, 25 μL 5 × sodium dodecyl sulfate sample loading buffer solution was added to the precipitation, and the supernatant was boiled for 10 min at 100 °C. Western blot analysis was performed with the secondary antibody to detect the interaction with eluted proteins.

### PHA-L staining

CFs were fixed with 4% paraformaldehyde for 20 min, blocked with Carbo-Free blocking solution for 30 min at room temperature to prevent non-specific binding, and then incubated with biotinylated PHA-L (1:200, Life Technologies, USA) in PBS for 30 min. After washing with PBST, ImmPACT Vector Red was applied for color development. The slides were then observed with a 60 × water objective by (Fluoview 1000, Olympus).

### Enzyme-linked immunosorbent assay (ELISA)

Human plasma samples were diluted with 1% bovine serum albumin and PBS containing 0.1% Tween-20 sample dilution buffer at 1:10. Then, 100 μL of the diluted plasma sample was added to a microtitration dish coated with 4G12 monoclonal antibody against Hp. The plate was incubated on a shaker at 750 rpm for 30 min. After washing five times with 300 μL washing buffer (PBS plus 0.5% Tent-20), 100 μL 4G12 monoclonal antibody conjugated to horseradish peroxidase (in dilution buffer) was added and placed on a shaker at 750 rpm for 1 h. After extensive washing as described above, 3, 3', 5, 5'-tetramethylbenzidine substrate was added, and the samples were oscillated at 750 rpm for 15 min. Finally, 100 μL stop solution (1 N HCl) was added, and the absorption was determined at 450 nm within 15 min.

### Statistics analysis

GraphPad Prism 8.0 software (GraphPad Software, La Jolla, USA) software was used to analyze the experimental data. All values are presented as the mean ± standard error of the mean (SEM). Comparison between two groups was performed using Student's *t* test. Comparisons between more than two groups were performed using one-way ANOVA, followed by post-hoc tests (with minimal differences). *P* < 0.05 was defined as the threshold for statistical significance.

## Results

### STZ-induced diabetic hearts exhibit excessive collagen deposition and upregulated CD147 expression

**Figure 1:**
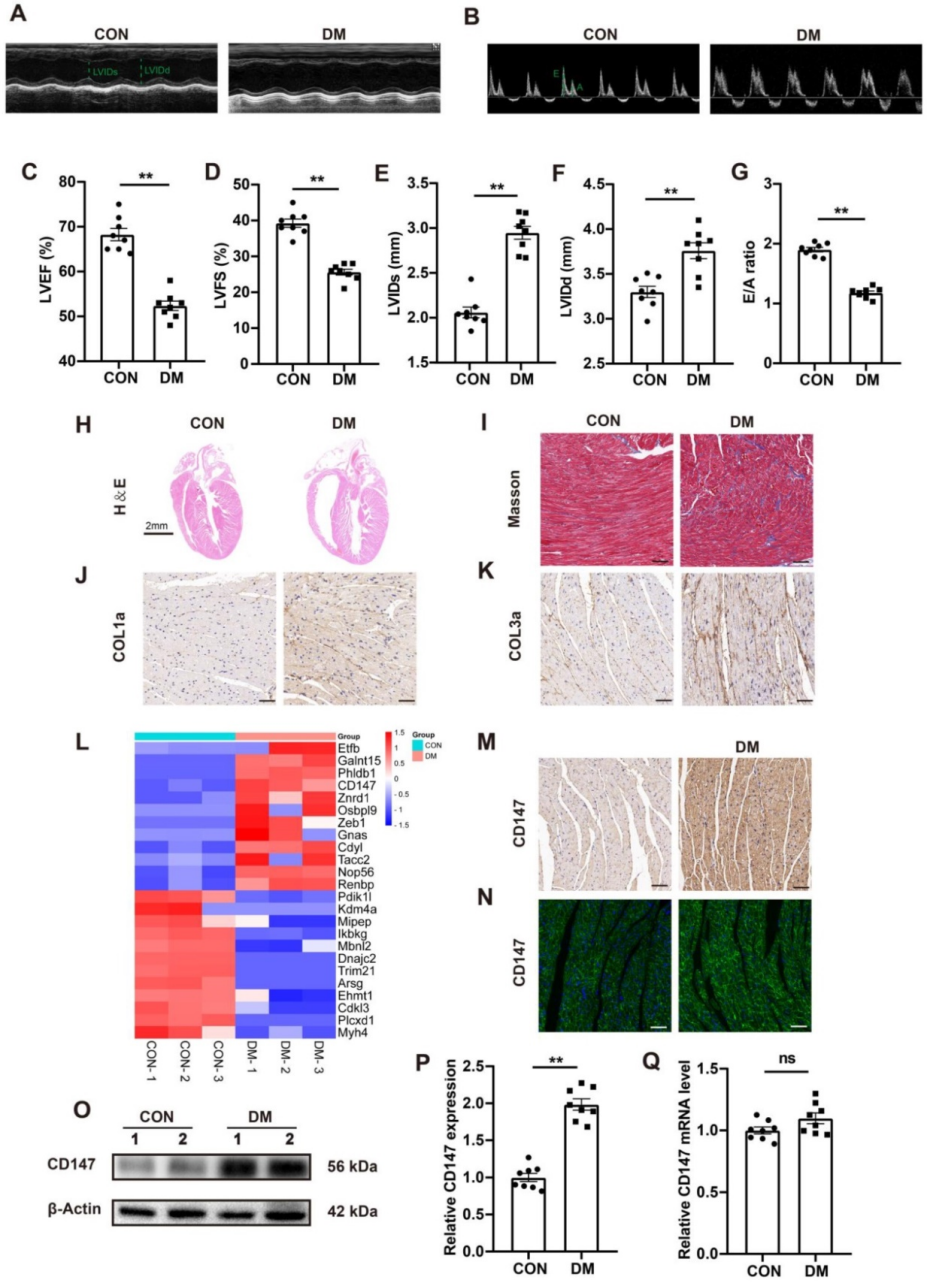
** STZ-induced diabetic hearts exhibit excessive collagen deposition and upregulated CD147 expression. (A, B)** Representative images of echocardiography; LVIDs, LVIDd, E/A ratio are marked (green). **(C-G)** Analysis of echocardiography data; n = 8 mice. **(H)** Representative H&E staining of hearts. Scale bar = 2 cm; n = 8 mice. **(I)** Representative Masson's trichrome staining of hearts. Scale bar = 30 µm; n = 8 mice. **(J, K)** Representative immunohistochemical staining of COL1a, COL3a in mice hearts. Scale bar = 30 µm; n = 8 mice. **(L)** Heatmap of Quantitative Proteomic Analysis of hearts; n = 6 mice. **(M, N)** Representative immunofluorescence and immunohistochemical staining of CD147 in mice hearts; n = 8 mice. **(O-Q)** Representative western blot and real-time PCR analysis of CD147 in mice hearts; n = 8 mice. Data represent as mean ± SEM. *p < 0.05, **p < 0.01.

Compared with control mice, the blood glucose level in STZ-induced diabetic mice was significantly increased 3 months after STZ injection, while the body weight and glucose tolerance were significantly decreased (Fig. S1A-C). Echocardiography showed considerable impairment in cardiac function in STZ-injected mice, as seen by decreased LVEF and LVFS and increased LVIDs and LVIDd (Fig. 1A, C-F). Moreover, mitral valve flow pattern analysis by doppler echocardiography also showed diastolic dysfunction in diabetic mice, and the E/A ratio decreased compared to that in control mice (Fig. 1B, G). Myocardial fibrosis is the main pathologic feature in DCM. HE and Masson's trichome staining analyses shown more extensive interstitial fibrosis and larger heart cavity in diabetic mice than in control mice. Furthermore, immunohistochemical staining and western blotting showed excessive collagen (COL1a and COL3a) deposition in the diabetic heart tissue (Fig. 1H-K; Fig. S1F, G). Quantitative proteomic analysis revealed upregulated expression of CD147, a transmembrane glycoprotein (Fig. 1L), in the diabetic hearts compared to control hearts. Immunohistochemical and IF staining analyses confirmed the above findings (Fig. 1M, N; Fig. SH). Western blotting also revealed upregulated CD147 expression in the diabetic heart tissue and HG-stimulated CFs (Fig. 1O, P; Fig. S1Q-L). However, the real-time PCR did not show significant difference of CD147 expression no matter in the tissues or cells (Fig. 1Q; Fig. S1M).

### Silencing CD147 alleviates cardiac dysfunction and fibrosis in diabetic hearts

**Fig 2:**
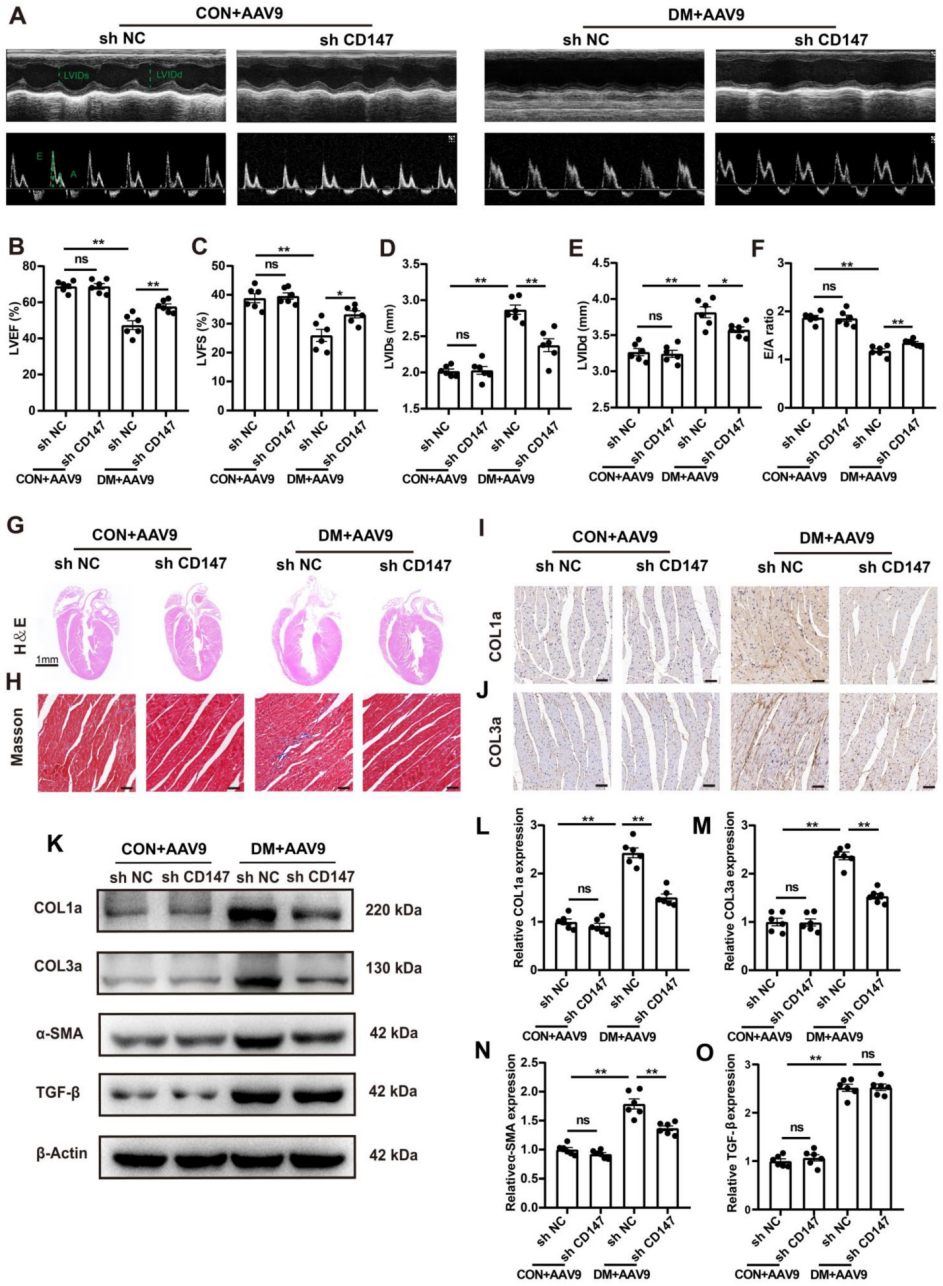
** Silencing CD147 alleviates cardiac dysfunction and fibrosis in diabetic hearts. (A)** Representative images of echocardiography; LVIDs, LVIDd, E/A ratio are marked (green). **(B-F)** Analysis of echocardiography data. (G) Representative H&E staining of hearts. Scale bar=1 mm; **(H)** Representative Masson's trichrome staining of hearts. Scale bar = 30 µm. **(I, J)** Representative immunohistochemical staining of COL1a, COL3a in mice hearts. Scale bar = 30 µm. **(K-O)** Representative western blot and analysis of COL1a, COL3a, α-SMA, TGF-β in mice hearts. n = 6 mice in each group. Data represent as mean ± SEM. *p < 0.05, **p < 0.01.

Whether CD147 regulates cardiac function in diabetes was investigated through loss of function assays of CD147 by employing AAV9-shCD147 (Fig. S2A). For the duration of AAV9-shCD147 *in vivo*, the virus began to have an effect at 16 weeks, and this effect peaked at 18 weeks and continued until 20 weeks (Fig. S2B, C). The immunohistochemical further results indicated that AAV9-shCD147 remarkably suppressed CD147 expression at 20 weeks, indicating successful virus injection and high efficiency of suppression (Fig. S2D, E). AAV9-shCD147 did not significantly affect blood glucose and body weight of control and model mice (Fig. S2F, G). However, the echocardiogram showed that when CD147 was downregulated in the heart tissue, cardiac function significantly improved and cardiac adverse remodeling in diabetic mice was alleviated, as seen by increased LVEF and LVFS and decreased E/A ratio, LVIDs and LVIDd (Fig. 2B-F). Moreover, HE and Masson's trichome staining showed less fibrotic area and greater heart cavity in diabetic mice with AAV9-shCD147 injection than control mice, highlighting the antifibrotic effects of targeting CD147 in diabetes (Fig. 2G, H; Fig. S2H, I). Immunohistochemical staining, western blotting, and real-time PCR analyses also showed less deposition of COL1a and COL3a and lower expression of fibrotic markers including COL1a, COL3a, and α-SMA in cardiac tissues upon CD147 knockdown (Fig. 2I-O; Fig. S2J-L). However, the protein and mRNA levels of TGF-β, a central mediator of fibrosis, did not change significantly in AAV9-shCD147 diabetic mice (Fig. 2O-S). This showed that CD147 knockdown did not affect the expression of TGF-β. CD147 knockdown in control hearts did not significantly affect cardiac function and fibrosis (Fig. 2A-O; Fig. S2D-L).

### CD147 is involved in HG-induced activation of CFs

**Figure 3:**
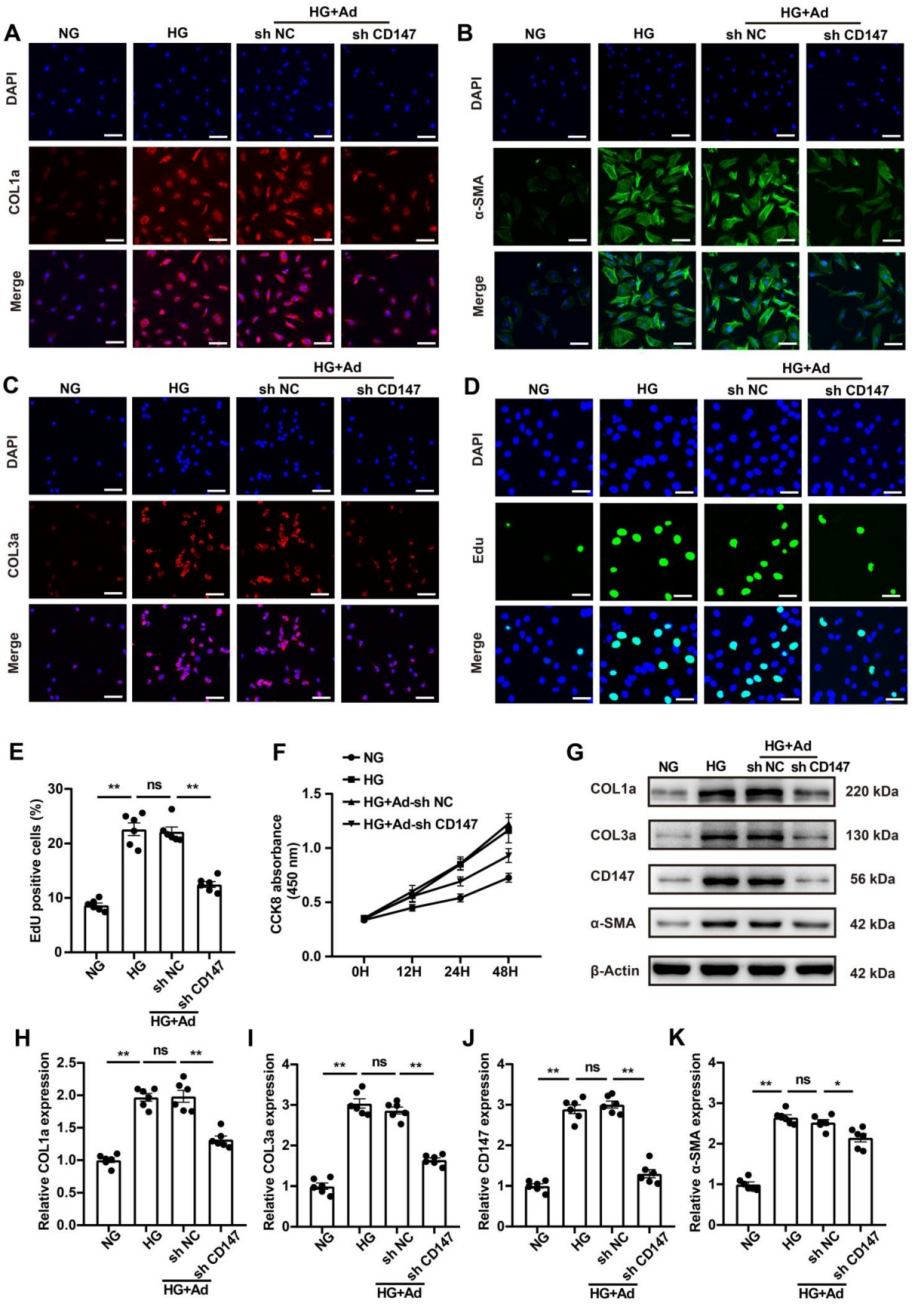
** CD147 is involved in HG-induced activation of CFs. (A-C)** Representative immunofluorescence images of COL1a (red), COL3a (red), α-SMA (green) in primary cardiac fibroblasts. Nucleus were stained with DAPI (blue). Scale bar=30 µm. **(D, E)** EdU assay by double staining with EdU (green) and DAPI (blue). Scale bar=15 µm. The quantification of EdU positive cells rates. **(F)** CCK8 assay to detect CFs proliferation at 0, 12, 24 and 48 h. **(G-K)** Representative western blot and analysis of COL1a, COL3a, CD147, α-SMA in primary cardiac fibroblasts. n = 6 wells in each group. Data represent as mean ± SEM. *p < 0.05, **p < 0.01.

Fibroblasts are the primary contributor to cardiac fibrosis, and HG is known to promote the activation and proliferation of fibroblasts 24, 25. HG (33 mM) treatment, simulating diabetes *in vitro*, significantly increased the expression levels of COL1a, COL3a, TGF-β, and α-SMA in CFs (Fig. 3A-C; G-K). Additionally, it remarkedly promoted the proliferation of CFs (Fig. 3D-F). Then we explored whether increased CD147 contributes to HG-induced activation and proliferation of fibroblasts using an *in vitro* model. Downregulation of CD147 in CFs by Ad-shCD147 significantly decreased the expression of COL1a, COL3a, TGF-β, and α-SMA as was seen by IF and western blot analyses (Fig. 3A-C; G-K). Furthermore, the induction effect of HG on CF proliferation was significantly reversed by CD147 shRNA (Fig. 3D-F). However, Flow cytometry and western blotting analysis showed that CD147 downregulation also did not affect the apoptosis of CFs and levels of cleaved-caspase-9 between control (5 mM) and HG (33 mM) group (Fig. S3A-D).

### CD147 activates TGF‑β downstream signaling in HG-induced CFs

**Figure 4:**
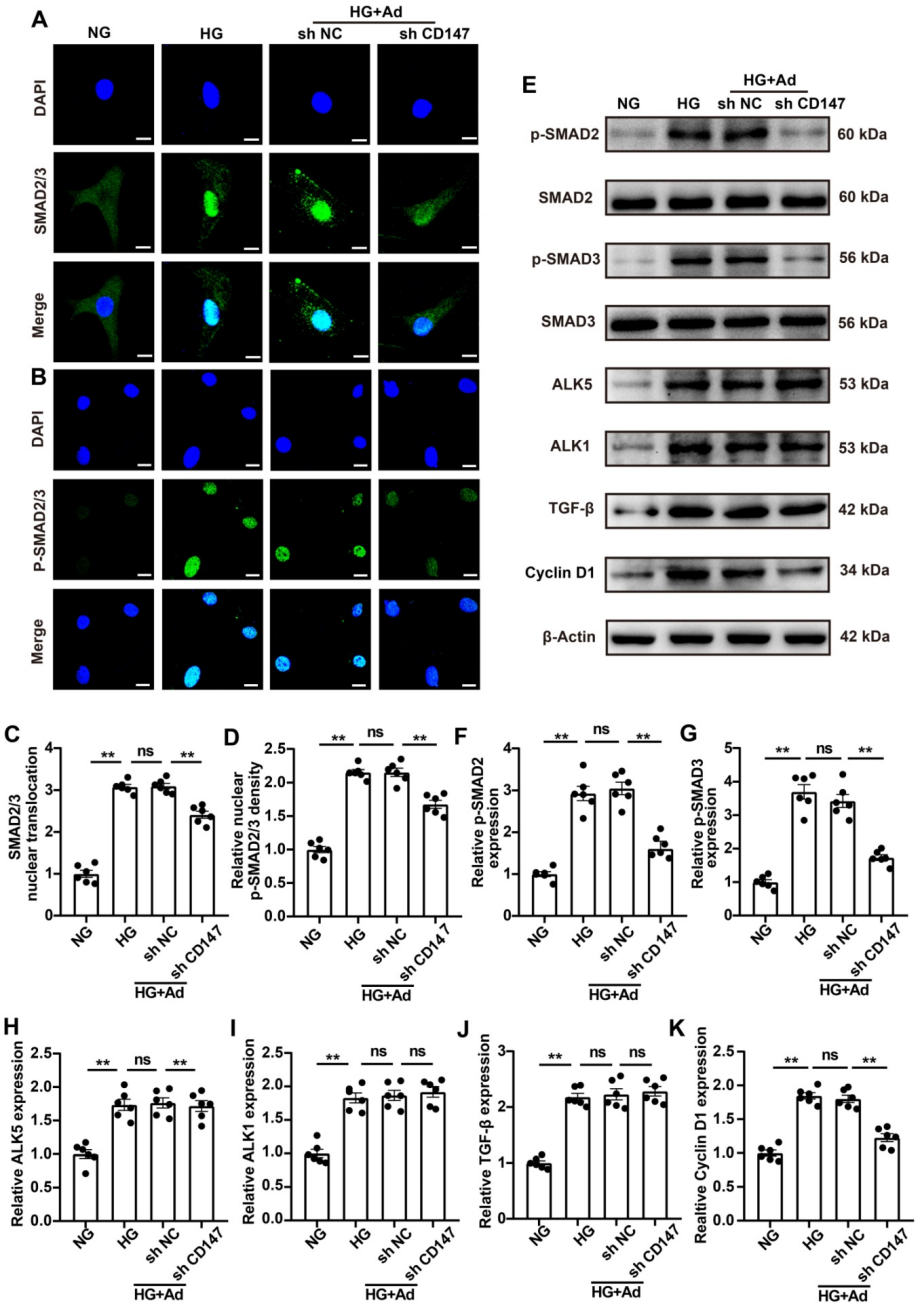
** CD147 activates TGF‑β downstream signaling in HG-induced CFs. (A, B)** Representative immunofluorescence images for co-localization of SMAD2/3 (green) and nucleus stained with DAPI (blue). Scale bar = 10 µm. Representative immunofluorescence images for co-localization of p-SMAD2/3 (green) and nucleus stained with DAPI (blue). Scale bar = 20 µm. **(C, D)** Quantification of SMAD2/3 nuclear translocation and p-SMAD2/3 nuclear density. (E-K) Representative western blot and analysis of p-SMAD2, p-SMAD3, ALK5, ALK1, TGF-β, Cyclin D1 in primary cardiac fibroblasts. n = 6 wells in each group. Data represent as mean ± SEM. *p < 0.05, **p < 0.01.

TGF-β is a “major” cytokine/growth factor that activates fibroblasts and promotes ECM production through canonical SMAD signaling in injured or diseased tissues 6, 7 .The molecular mechanisms by which CD147 regulates the HG-induced activation and proliferation of CFs were investigated by incubating the CFs in media containing different concentrations of glucose (5 mM and 33 mM) for 2 days to mimic diabetes *in vitro* followed by transfection with Ad-sh CD147 or Ad-sh NC. IF and western blotting analyses showed that HG treatment activated the TGF-β/SMAD signaling pathway (Fig. 4A-I). Knocking down CD147 profoundly decreased the nuclear translocation of SMAD2/3, downstream of TGF-β, and suppressed the levels of proteins including p-SMAD2 and p-SMAD3 compared with control (Fig. 4A-G). Cyclin D1, a target of p-SMAD2/3, regulates cell cycle and promotes cell proliferation; its level was also decreased in the HG-treated group in the presence of Ad-shCD147 (Fig. 4E, J). However, downregulation of CD147 failed to reverse the increased the expression of TGF-β, ALK5 (TGFβ-RI), and ALK1 (TGFβ-RII) after HG treatment (Fig. 4A-F). These findings highlighted the role of CD147 in regulating the activation of TGF‑β signaling, independent of affecting the expression of TGF-β and its receptors.

### CD147 facilitates ALK5 activation and endocytosis to regulate HG-induced SMAD2/3 phosphorylation and nuclear translocation

The mechanism by which CD147 modulates the activation of TGF-β signaling was investigated by LC-MS/MS analysis of heart tissues, which showed that CD147 was able to interact with a 50 kDa subunit of TGF-β receptor type I (ALK5), and it was selected for further study (Fig. S4A).

**Figure 5:**
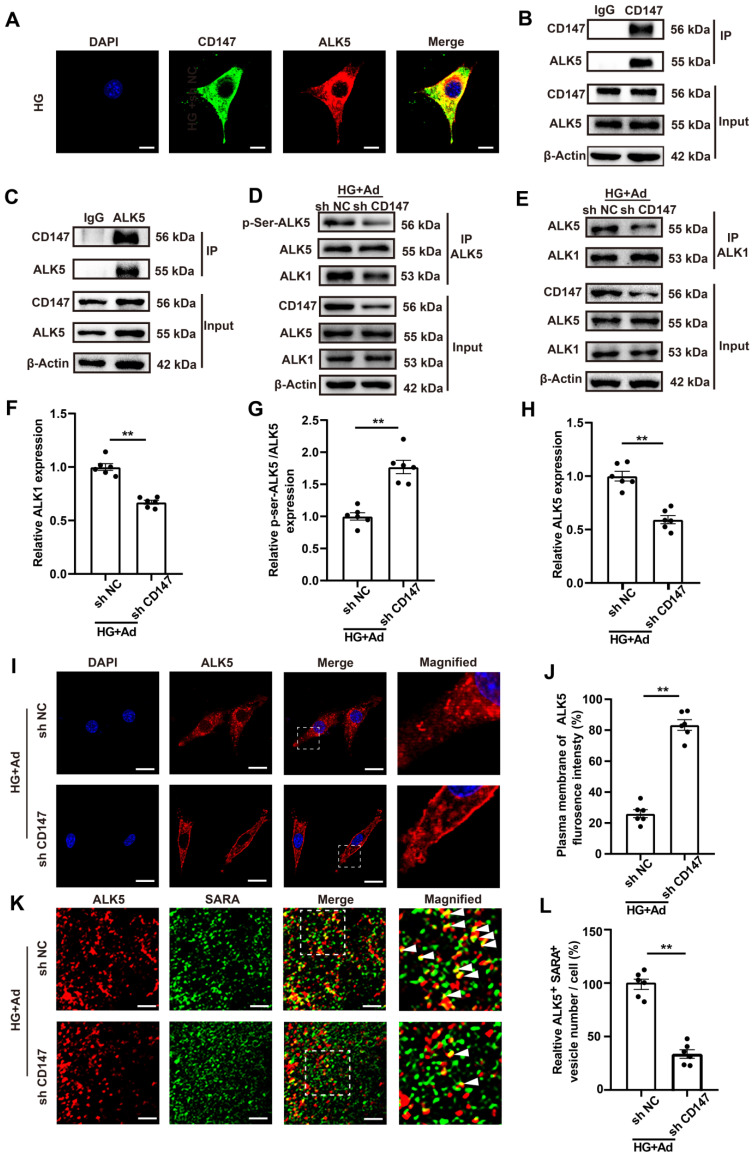
** CD147 facilitates ALK5 activation and endocytosis to regulate HG-induced SMAD2/3 phosphorylation and nuclear translocation. (A)** Representative immunofluorescence images for co-localization of CD147 (green), ALK5 (red) and DAPI (blue) in primary cardiac fibroblasts. Scale bar = 10 µm. **(B, C)** Interaction between CD147 and ALK5 was demonstrated by Co-IP. **(D-H)** Co-IP of ALK5 and ALK1 in HG-treated sh-NC and sh CD147 primary cardiac fibroblasts. **(I, J)** Representative immunofluorescence images for ALK5 (red) internalization and quantification of ALK5 fluorescence density on the cell membrane. Nucleus were stained with DAPI (blue). Scale bar = 10 µm. **(K, L)** Representative immunofluorescence images and quantification for co-localization of ALK5 (red), SARA (green) and DAPI (blue) in primary cardiac fibroblasts. Scale bar = 1 µm. n = 6 wells in each group. Data represent as mean ± SEM. *p < 0.05, **p < 0.01.

The interaction between CD147 and ALK5 in diabetic heart tissues was investigated through several subsequent experiments. IF analysis showed remarkable colocalization of CD147 and ALK5 in CFs (Fig. 5A). Co-IP assays showed that CD147 antibody was able to pull down ALK5 protein and vice versa (Fig. 5B, C). However, the colocalization of CD147 and ALK5 did not significantly differ between normal (5 mM) and HG (33 mM) treatments (Fig. S4B, C). ALK1 first binds with TGF-β and then recruits and phosphorylates the kinase domain of ALK5, which then phosphorylates SMAD proteins. Knocking down CD147 decreased the interaction of ALK1 and ALK5 and the phosphorylation of ALK5 but did not affect the expression of both as demonstrated by Co-IP analysis (Fig. 5D-H). Phosphorylated ALK5 leaves the cell membrane and internalizes into the early endosome termed 'endocytosis', which is equally important for SMAD 2/3 phosphorylation. IF analysis further confirmed that HG promoted ALK5 endocytosis into the intracellular compartment, which could be prevented by CD147 knockdown (Fig. 5I, J). The SMAD anchor for receptor activation (SARA) located in early endosomes is required for SMAD2/3 phosphorylation by ALK5 and can be used as a marker of early endosomes. We next investigated the spatial location of ALK5 and SARA and the effect of CD147 knockdown on the co-localization. HG increased the colocalization of ALK5 and SARA as seen by more positive yellow dots, while CD147 knockdown significantly decreased ALK5-SARA colocalization (Fig. 5K, L). Overall, our data suggested that CD147 may act as a scaffold to facilitate ALK5 activation and endocytosis, thus eventually activates TGF-β downstream signaling.

### HG promotes CD147 glycosylation and suppresses its ubiquitin-proteasomal degradation

**Figure 6:**
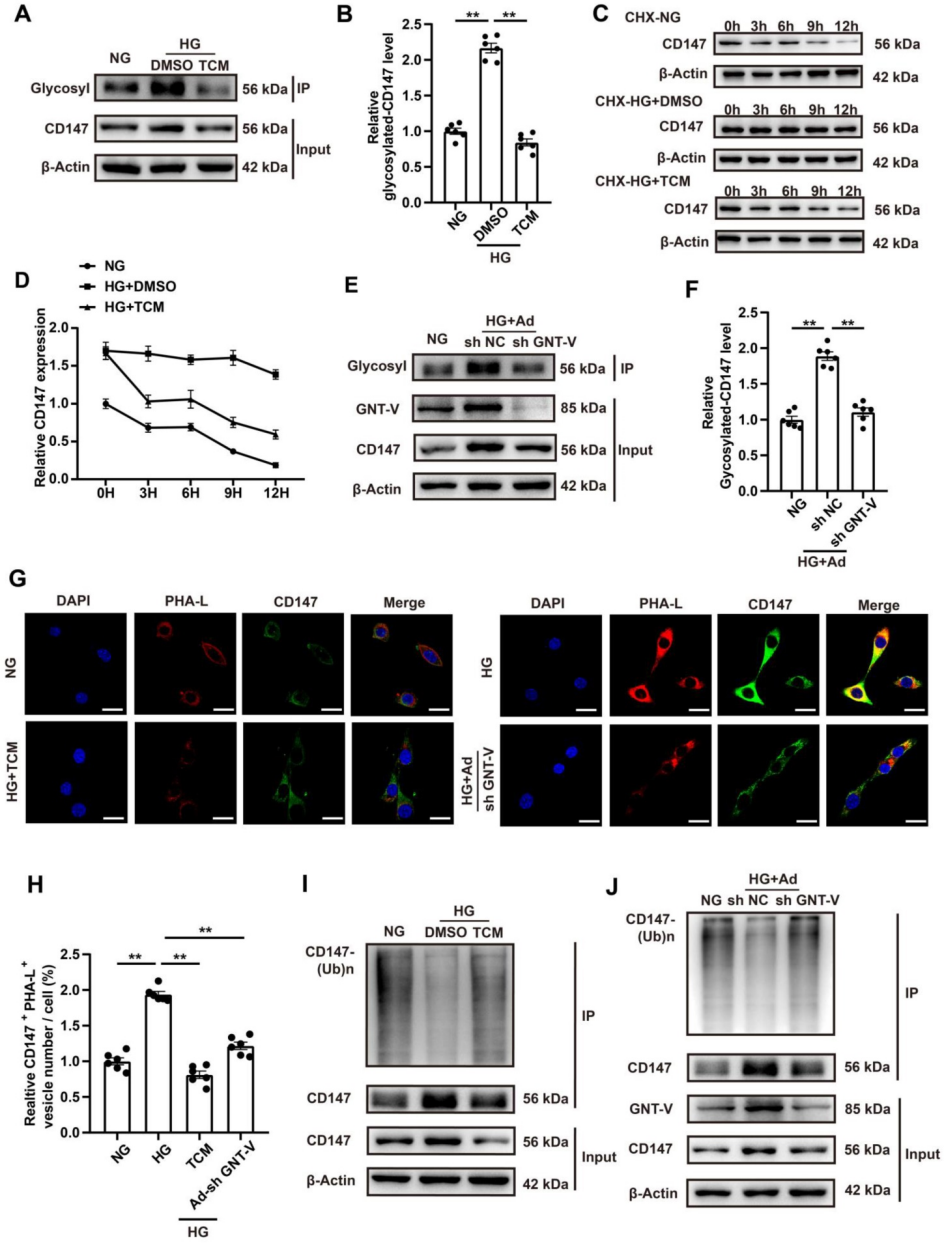
** HG promotes CD147 glycosylation and suppresses its ubiquitin-proteasomal degradation. (A, B)** Glycosylation level of CD147 were determined in HG-treated primary neonatal cardiac fibroblasts after TCM stimulation. **(C, D)** Time-dependent CD147 protein level was determined by western-blotting in CHX-treated primary cardiac fibroblasts. **(E, F)** Glycosylation level of CD147 were determined in HG-treated primary neonatal cardiac fibroblasts after GNT-V knock down via ad-sh RNA. **(G, H)** Representative immunofluorescence images and quantification for co-localization of PHA-L (red), CD147 (green) and DAPI (blue) in primary cardiac fibroblasts. Scale bar = 20 µm. **(I, J)** IP assay and western blot analysis determined CD147 protein and ubiquitylation level in the presence of HG and MG-132. n = 6 wells in each group. Data represent as mean ± SEM. *p < 0.05, **p < 0.01.

The mechanism by which HG regulated CD147 expression was evaluated by determining the transcription and degradation of CD147. Western blotting and real-time PCR data showed that HG treatment remarkably increased the expression of CD147 at the protein level but not the mRNA level, indicating that CD147 is regulated at a non-transcriptional level (Fig. 1O-Q; Fig. S1M-P). Since CD147 is a transmembrane glycoprotein that can be modified post-translationally (e.g., glycosylation), we determined the degree of CD147 glycosylation. As expected, HG significantly increased CD147 glycosylation, which was reversed by tunicamycin (TCM), a protein glycosylation inhibitor (Fig. 6A, B; H, I). Cycloheximide (CHX), as a blocker of protein synthesis, was used to explore the relationship between glycosylation and protein stability. HG treatment stabilized CD147 protein levels in CHX-treated cells over time, which was reversed by TCM (Fig. 6C, D). N-Acetylglucosaminyltransferase V (GNT-V) is a key enzyme required for CD147 glycosylation in cancer metastasis, and we investigated whether GNT-V might be responsible for the increased glycosylation of CD147 in CFs upon HG treatment. Co-IP assay confirmed the interaction of CD147 and GNT-V *in vitro*, and GNT-V expression was also further increased after HG treatment (Fig. S5A; Fig. 6E). Knocking down GNT-V significantly reduced the protein and glycosylation levels of CD147 in HG-treated CFs (Fig. 6E-H). MG-132, a potent and reversible proteolysis inhibitor, significantly decreased the ubiquitination of CD147 in HG-induced CFs as demonstrated by Co-IP analysis (Fig. 6I, J). This effect was reversed by knocking down both TCM and GNT-V, which also dramatically reduced the expression level of CD147, COL3a, and α-SMA in HG-treated CFs (Fig. S5B-F). More importantly, in diabetic mice, interfering with the expression of GNT-V reduced the glycosylation level of CD147, which increased its ubiquitination level, and thus decreased collagen accumulation and cardiac dysfunction in the diabetic state (Fig. S5G-W). Overall, these data suggested that HG increases the expression of GNT-V, which then promotes CD147 glycosylation and prevents its ubiquitin-proteasomal degradation.

### CD147 overexpression in control mice mimicked diabetes-induced cardiac fibrosis

**Figure 7:**
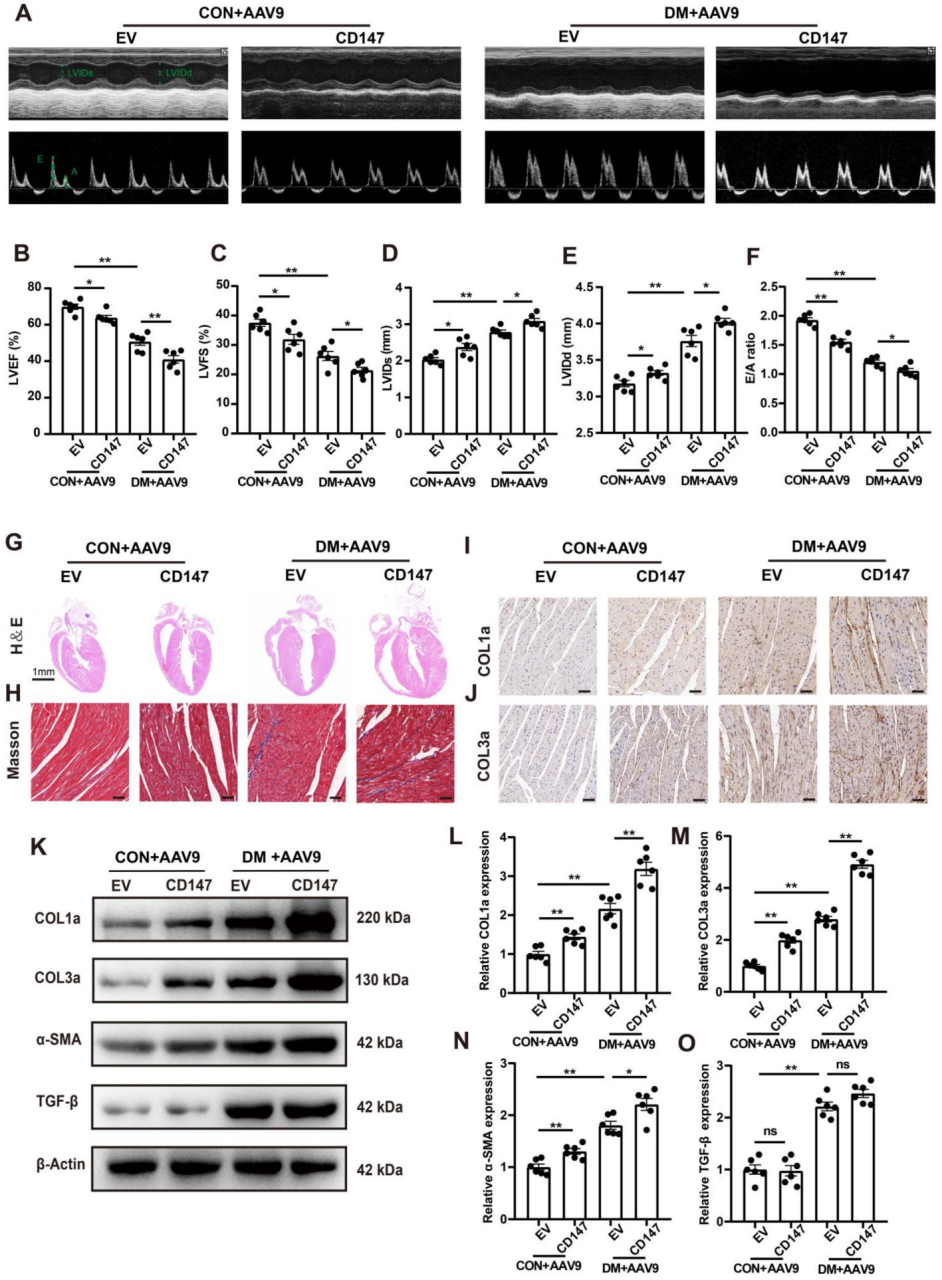
** CD147 overexpression in control mice mimicked diabetes-induced cardiac fibrosis. (A)** Representative images of echocardiography; LVIDs, LVIDd, E/A ratio are marked (green). **(B-F)** Analysis of echocardiography data. **(G)** Representative H&E staining of hearts. Scale bar = 1 mm. **(H)** Representative Masson's trichrome staining of hearts. Scale bar = 30 µm. **(I, J)** Representative immunohistochemical staining of COL1a, COL3a in mice hearts. Scale bar = 30 µm. **(K-O)** Representative western blot and analysis of COL1a, COL3a, α-SMA, TGF-β in mice hearts. n = 6 mice in each group. Data represent as mean ± SEM. *p < 0.05, **p < 0.01.

Whether CD147 overexpression could exacerbate the cardiac dysfunction and fibrosis in control mice was evaluated through intracardiac injection of AAV9-CD147 to increase CD147 expression in the cardiac tissues (Fig. S6A). For the duration of AAV9-CD147 *in vivo*, the virus began to have an effect at 16 weeks, and this effect peaked at 18 weeks and continued until 20 weeks (Fig. S6B, C). The immunohistochemical further results indicated that AAV9-CD147 remarkably elevated CD147 expression at 20 weeks, indicating successful virus injection and high efficiency of suppression (Fig. S6D, E). AAV9-CD147 did not significantly affect blood glucose and body weight both in control and diabetic mice (Fig. S6F, G). Echocardiography analysis showed that exogenic CD147 in control mice dramatically impaired systolic and diastolic function as seen by lower LVEF and LVFS and higher E/A ratio, LVIDs and LVIDd compared to control mice treated with AAV9-EV (Fig. 7A-F). HE and Masson's trichome staining confirmed the adverse cardiac remodeling caused by exogenic CD147 in control mice (Fig. 7G, H; Fig. S6H, I). Immunohistochemical staining also showed significantly elevated COL1a and COL3a deposition in control mice treated with AAV9-CD147 compared with the deposition levels seen in mice treated with AAV9-EV (Fig. 7I, J; Fig. 7N, O). The mRNA and protein levels of COL1a, COL3a, and α-SMA were notably increased after exogenic CD147 injection (Fig. 7K-O; Fig. S6J-L). As expectedly, TGF-β expression was not affected by CD147 overexpression (Fig. 7K, O). These results also indicated that CD147 overexpression exacerbated the cardiac function and promoted cardiac fibrosis in diabetic mice (Fig. 7A-O; Fig. S6D-L). More importantly, to investigate the effect of CD147 on cardiomyocytes, adeno-associated virus of AAV9-cTnT-CD147 were prepared and continuously expressed in mouse heart for one month. The CD147 gene is driven by cTnT promoter to ensure cardiomyocyte specific expression. The AAV9-cTnT-CD147 in control mice failed to mimick diabetes-induced dysfunction (Fig. S7A-F). The results of immunohistochemistry and western-blot further showed that there was no obvious collagen deposition in the AAV9-cTnT-CD147 group (Fig. S7G-V), which further confirmed that CD147 plays a pro-fibrotic role through cardiac fibroblasts.

### CD147 regulation and cardiac function in the serum and cardiac tissues of patients with diabetes

**Figure 8:**
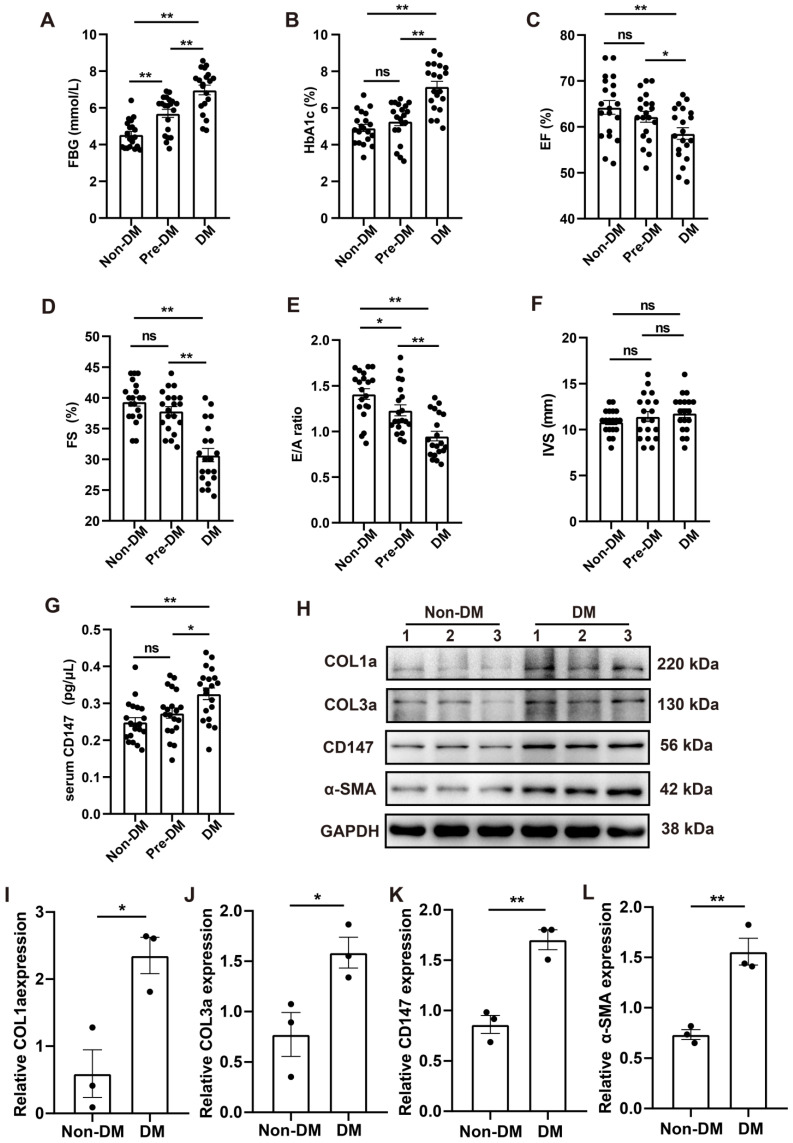
** CD147 regulation and cardiac function in the serum and cardiac tissues of patients with DM. (A-F)** FBG, HbA1c, EF, FS, E/A ratio, IVS are compared among non-diabetic and diabetic patients. n=20 per group. **(G)** ELISA assay to determine CD147 levels in serum of patients. n=20 per group. **(H-L)** Representative western blot and analysis of COL1a, COL3a, CD147, α-SMA in atrial tissue from non-diabetic or diabetic individuals. n=3 per group. Data represent as mean ± SEM. *p < 0.05, **p < 0.01.

Clinical data including echocardiography and clinical samples from DM, Pre-DM and age-matched subjects without DM undergoing open-heart valve replacement surgery were analyzed. Baseline parameters of the three groups are shown in Table S2. Fasting blood glucose (FBG) and glycosylated hemoglobin (HbA1c) levels were markedly higher in patients with DM than in those without diabetes; The level of FBG in Pre-DM group was intermediate between DM and non-DM group and there is no significant difference in the HbA1c level between Pre-DM and non-DM group (Fig. 8A, B). Echocardiography data showed impaired systolic and diastolic function in patients with DM as evidenced by decreased LVEF and LVFS and increased E/A ratio. Pre-DM patients exhibit some degree of diastolic dysfunction compared with non-DM group; however, the interventricular septum thickness did not increase in the Pre-DM and DM patients (Fig. 8C-F). Human CD147 ELISA showed that the serum level of CD147 the increased to some extent in Pre-DM group although there was no statistical difference and was significantly higher in patients with DM than in those without DM. (Fig. 8G). Furthermore, COL1a, COL3a, CD147, and α-SMA levels were higher in the myocardium of patients with DM than in those without DM, consistent with our preclinical data (Fig. 8H-L).

## Discussion

Cardiac fibrosis is a hallmark complication seen in patients with DM, which leads to heart stiffness and impaired heart function 3, 23, 25. However, the pathogenesis of cardiac fibrosis in DCM remains unclear. Herein, we demonstrated that CD147 played an important role in cardiac fibrosis in DM. First, we detected excessive collagen deposition and upregulated CD147 expression both in diabetic mouse hearts and clinical samples. Second, *in vivo* and *in vitro* assays revealed that CD147 knockdown alleviated cardiac fibrosis and prevented fibroblast activation in DCM by targeting the TGF-β signaling pathway. Third, HG significantly increased the N-glycosylation of CD147 by upregulating GNT-V, which prevented its ubiquitin-proteasomal degradation. Then stabilized CD147 can directly bind to ALK5, promoting ALK5 activation and endocytosis to induce SMAD2/3 phosphorylation and nuclear translocation. Finally, cardiac-specific CD147 overexpression in control mice mimicked diabetes-induced cardiac fibrosis, leading to cardiac damage. This study, for the first time, demonstrates the vital roles of CD147 in mediating cardiac fibrosis in DM. As such, CD147 may be a new therapeutic target for DCM.

Diabetes-associated cardiac fibrosis involves the activation of CFs in response to hyperglycemia, leading to adverse cardiac remodeling with excessive collagen deposition 25, 26. In this study, CD147 expression was remarkably increased and severe cardiac fibrosis occurred three months after STZ injection in the diabetic heart tissues of mice. Cardiac-specific silencing of CD147 alleviated myocardial dysfunction and attenuated myocardial fibrosis in diabetic mice, whereas its overexpression aggravated myocardial dysfunction with excessive collagen accumulation in control or diabetic mice. Previous studies have also indicated the profibrotic effect of CD147 in interstitial lung diseases, liver cirrhosis, and diabetic nephropathy 19, 27, 28. *In vitro* data consistently demonstrated that CD147 knockdown significantly inhibited the HG-indued activation of cardiac fibroblasts as seen by the downregulated expression of fibrotic markers and reduced proliferation of CFs. Overall, our study revealed a new phenomenon that imbalanced synthesis and degradation of collagen induced by increased CD147 contributes to the development of DCM through the activation of CFs.

TGF-β has been extensively studied as a fibrogenic growth factor in cardiac fibrosis 29. HG can increase the transcription of TGF-β, resulting in activated TGF-β signaling and transduction of downstream SMAD-dependent pathways 6, 7, 25. Although CD147 can directly induce TGFβ expression in multiple cells including hepatic stellate cells, hepatocellular carcinoma, and tubular epithelial cells 18, 19, 30, the potential relationship between CD147 expression and TGF-β receptor, especially in the heart, has not been studied. For the first time, we demonstrate that CD147 directly binds to ALK5 but does not influence the expression of TGF-β. ALK5, also known as TGF-β receptor I, binds to TGF-β in the plasma membrane, which forms a heterodimeric complex upon activation by ALK1. ALK5 is then internalized into early endosomes, where SARA interacts with SMAD2/3 and promotes its phosphorylation and nuclear translocation 7, 25. We further found that CD147 promotes ALK5 activation on the cell membrane and its subsequent endocytosis in the cytoplasm, which shows that CD147 acts as a non-classical upstream regulator of TGF-β receptor and related signaling pathways. Because TGF-β signaling can also modulate other physiological processes such as angiogenesis, immunosuppression, and carcinogenesis 7, 31, 32, blocking of the ligand TGF-β or its receptors can lead to severe inflammation shortly after birth and fetal death 31, 33. In our study, CD147 only participates in the regulation of fibrogenic function for ALK5, suggesting that CD147 might be a more feasible target for regulation of downstream TGF-β signaling and fibrosis diseases.

Glycosylation is a common post-translational modification that occurs in diabetes. Elevated glycosylation of cardiac ECM components promotes ECM deposition 34, 35. In this study, we demonstrated, for the first time, that N-glycosylation of CD147 accelerated the process of fibrosis in the diabetic heart by affecting CD147 protein stability. Specifically, increased GNT-V-mediated N-glycosylation of CD147 under the HG condition, which further impeded its ubiquitin-proteasomal degradation. As a result, cumulative CD147 lead to cardiac fibrosis in the diabetic heart. In contrast, GNT-V knockdown inhibited the HG-induce N‐glycosylation of CD147 and cardiac fibrosis. Previous works have demonstrated N‐glycosylation of CD147 mediated by GNT-V has the high activity to stimulate the production of MMP-2 and MMP-9, thus lead to degradation of type IV collagen and promoted tumor cell invasion and metastasis in multiple types of cancer 36. Since the ECM of heart tissue is mainly composed of type I and III collagen. Therefore, our study provided a different and deeper understanding of how glycosylation impacts CD147 stability to mediate collagen I and III deposition rather than activity. Interestingly, we also found a significant increase of GNT-V level in diabetic heart. Abnormally elevated glycosylation and GNT-V provide both enzymes and substrates for CD147 to be over-glycosylated in the diabetic heart, leading to aberrant CD147 accumulation and irreversible impairment of cardiac function. These data suggested that constant CD147 glycosylation in high-glucose environment acts as a pivotal contributor for cardiac function altering from compensated to decompensated states.

Consistent with other reports 37-39, we observed minor impaired diastolic dysfunction in Pre-DM patients and evident systolic dysfunction in DM patients as seen by decreased LVEF and LVFS and increased E/A ratio. DM is closely related to myocardial fibrosis and significantly increases the risk of ejection fraction-reserved heart failure 40. A direct biopsy study of diabetic myocardial specimens also showed that increased myocardial interstitial fibrosis causes left ventricular hypertrophy 41. In our study, patients with DM had more collagen accumulation and elevated α-SMA expression. Moreover, the CD147 level was increased in the serum and myocardium of these patients. It should be noted that the expression of CD147 increased to some extent in the Pre-DM patients although there was no statistical difference, which means that alteration of CD147 expression may be more closely related to higher blood glucose levels and systolic dysfunction. All clinical data were consistent with our *in vivo* and *in vitro* experimental results. These translational results suggest that our study has a good application value and prospect in the clinical treatment of cardiac fibrosis in patients with diabetes.

There are several limitations to our study. First, CF-specific CD147-knockout mice were not used in this study. In order to logically support our results, a series of vitro experiments on CFs were conducted. From another side, cardiac-specific CD147 overexpression was conducted by injecting AAV9-CD147 into hearts. Second, details regarding ALK5 activation and endocytosis were not evaluated. Third, the relationship between CD147 glycosylation and ubiquitin degradation pathway needs further research.

In conclusion, we have proposed a novel mechanism by which CD147 promotes ALK5 activation and endocytosis in early endosomes, promoting cardiac fibrosis in DCM through enhanced downstream SMADs signal transduction. HG promotes CD147 glycosylation, which reduces its ubiquitylation, which further leads to collagen accumulation and cardiac dysfunction in the diabetic heart. The findings of this study may facilitate the development of potential therapeutic strategies based on CD147 for DCM.

## Supplementary Material

Supplementary figures.

## Figures and Tables

**Figure 9:**
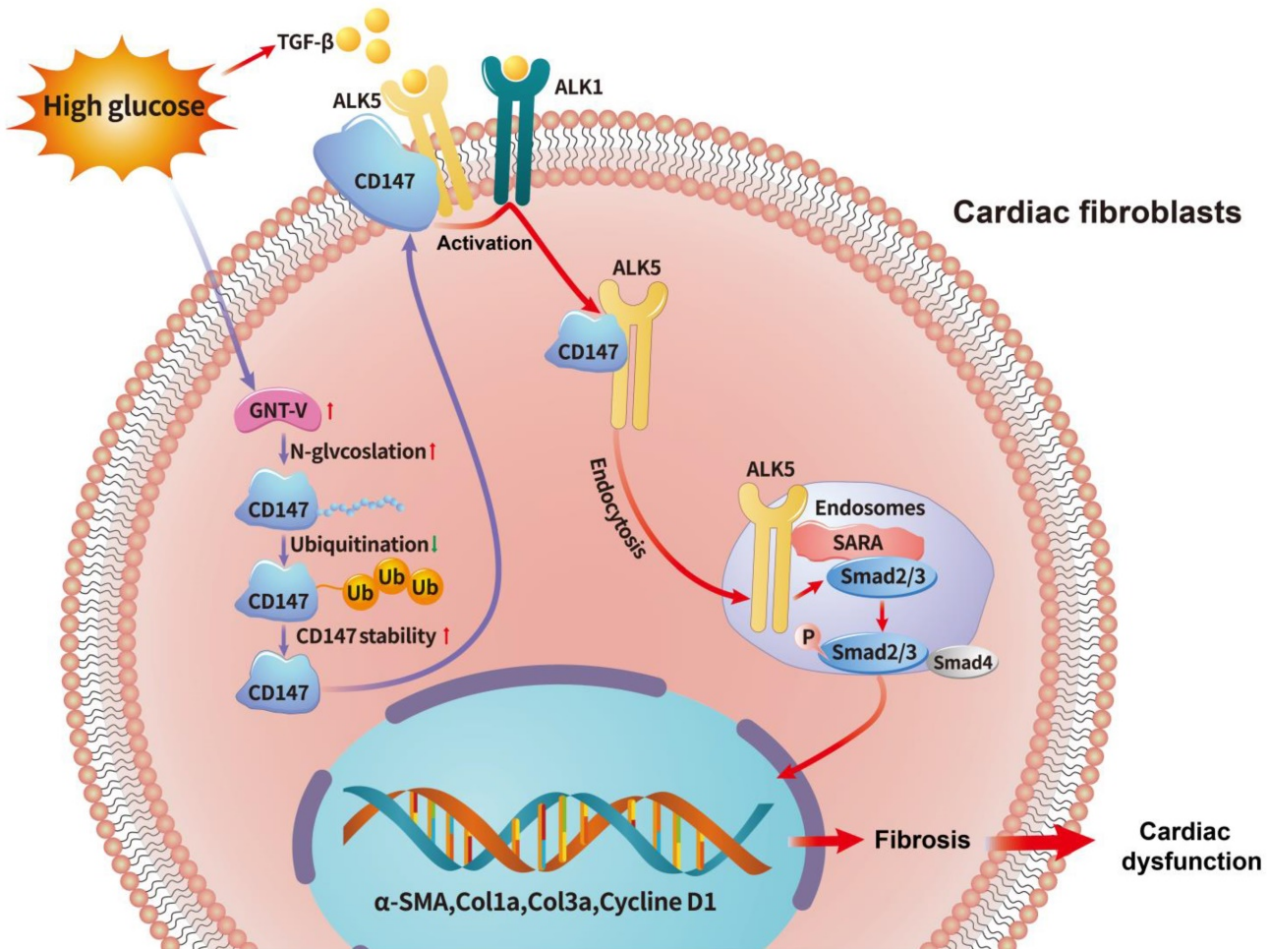
** Schematic diagram illustrating the mechanism by which N-glycosylation-mediated CD147 accumulation induces cardiac fibrosis in the diabetic heart by activating ALK5.** Diabetes (high-glucose) significantly increased the N-glycosylation of CD147 by upregulating GNT-V, which prevented its ubiquitin-proteasomal degradation. Stabilized CD147 can directly bind to ALK5, promoting ALK5 activation and endocytosis to induce SMAD2/3 phosphorylation and nuclear translocation. Silencing CD147 inhibits TGF‑β downstream signaling in HG-induced primary cardiac fibroblasts, which alleviates cardiac dysfunction and fibrosis in diabetic hearts.

**Table 1:** Primer sequences

Gene	Forward primer sequences (5'-3')	Reverse primer sequences (5'-3')
β-Actin	CATTGCTGACAGGATGCAGAAGG	TGCTGGAAGGTGGACAGTGAGG
COL1a	TCCTGACGCATGGCCAAGAA	CATAGCACGCCATCGCACAC
COL3a	GAAAAAACCCTGCTCGGAATT	ATCCATCTTGCAGCCTTG
CD147	CGTCATTATATCCACGCCTGAGCTG	GCGCACATTCTTGTCCTTGTCATTC
α-SMA	GCTATGCTCTGCCTCATGCC	CACGCTCAGCAGTAGTCACGAA
TGF-β	GGACTCTCCACCTGCAAGAC	CTCTGCAGGCGCAGCTCTG

## Abbreviations

CD147: cluster of differentiation 147
DCM: diabetic cardiomyopathy
CF: cardiac fibroblast
COL1a: collagen Ia
COL3a: collagen IIIa
TGF-β: transforming growth factor-β
ALK1: activin receptor-like kinase 1
ALK5: activin receptor-like kinase 5
SARA: SMAD anchor for receptor activation
TCM: tunicamycin
ECM: extracellular matrix
STZ: streptozotocin
α-SMA: α smooth muscle actin
siRNA: small interference RNA
AAV9: adeno-associated virus 9
Co-IP: co-immunoprecipitation
Real-time PCR: real time-polymerase chain reaction
NG: normal glucose
HG: high glucose
E/A ratio: ratio of E wave to A wave
LVIDd: left ventricle internal dimension at end-diastole
LVIDs: left ventricle internal dimension at end-systole
DM: diabetes mellitus
Pre-DM: prediabetes mellitus
IF: immunofluorescence
EdU: 5-ethynyl-2′-deoxyuridine
