# Supplementary material for: N-glycosylation-mediated CD147 accumulation induces cardiac fibrosis in the diabetic heart through ALK5 activation
Source: Int J Biol Sci. 2023 Jan 1;19(1):137–55. doi: 10.7150/ijbs.77469 (PMC9760447; doi:10.7150/ijbs.77469)
Supplement: Supplementary file 1 — Supplementary figures. [file ijbsv19p0137s1.pdf]

Supporting File

N-glycosylation-mediated CD147 accumulation induces cardiac fibrosis in the diabetic heart through ALK5 activation

Mingchuan Liu [1,#], Tingwei Peng [1,#], Lang Hu [1,#], Min Wang [2], Dong Guo [1], Bingchao Qi [1], Gaotong Ren [1], Di Wang [1], Yunqing Li [3], Liqiang Song [2], Jianqiang Hu [1,*], Yan Li [1,*]

Antibody	Target species	Working dilutions	Supplier	Catalog numbers
COL1a	Rabbit	WB: 1:2000<br>IF: 1:200<br>IHC: 1:100	Abcam, Cambirge, UK	ab138492
COL3a	Rabbit	WB: 1:1000<br>IF: 1:200<br>IHC: 1:100	Servicebio, Wuhan, China	GB111629
CD147	Rabbit	WB: 1:1000<br>IF: 1:200<br>IHC: 1:50<br>IP: 1:100	Abmart, Shanghai,China	T56711
α-SMA	Rabbit	WB: 1:2500<br>IF: 1:200	Proteintech, Wuhan, China	14395-1-AP
TGF-β	Rabbit	WB: 1:2000	Proteintech, Wuhan, China	21898-1-AP
ALK5	Mouse	WB: 1:1000	Santa Cruz, Dallas, USA	sc-101574

		IF: 1:200		
		IP: 1:100		
ALK1	Mouse	WB: 1:1000	Santa Cruz, Dallas, USA	sc-101556
		IP: 1:100		
SMAD2	Rabbit	WB: 1:1000	Proteintech, Wuhan, China	12570-1-AP
		IF: 1:100		
P-SMAD2	Rabbit	WB: 1:1000	CST, Boston, USA	18338S
		IF: 1:100		
SMAD3	Rabbit	WB: 1:1000	CST, Boston, USA	9523S
		IF: 1:100		
P-SMAD3	Rabbit	WB: 1:1000	CST, Boston, USA	9520S
		IF: 1:100		
CyclineD1	Rabbit	WB: 1:2000	Proteintech, Wuhan, China	610186-1-Ig
β-Actin	Mouse	WB: 1:5000	Proteintech, Wuhan, China	66009-1-Ig
GNT-V	Mouse	WB: 1:1000	Abcam, Cambirge, UK	ab87977
		IP: 1:100		
SARA	Rabbit	IF: 1:100	Abmart, Shanghai,China	T510680

Supplementary Table 1. The primary antibodies used for western blotting, immunoprecipitation, immunohistochemistry and immunofluorescence

**Items**	**Non-DM (n=20)**	**Pre-DM (n=20)**	**DM (n=20)**
Age（years）	72.15±10.06	66.90±10.06	70.85±9.22
Male %（n）	13（65%）	11 (55%)	11（55%）
Female %（n）	7（35%）	9 (45%)	9 (45%)
BMI（kg/m^2）	22.73±2.60	23.44±2.91	22.42±2.21
SBP (mmHg)	117.70±10.28	122.10±12.03	115.90±11.26
DBP (mmHg)	73.00±9.29	71.95±10.01	70.20±9.70
TG (mmol/L)	1.00±0.38	0.99±0.33	0.89±0.28
TC (mmol/L)	4.03±0.83	4.47±0.62	4.21±0.84
HDL-C (mmol/L)	1.35±0.57	1.16±0.27	1.46±0.57
LDL-C (mmol/L)	2.90±0.67	2.85±0.56	3.24±0.75
FBG (mmol/L)	4.54±0.74	$5.70\pm1.00^{**}$	$6.97\pm1.15^{\#\#\blacktriangle\blacktriangle}$
HbA1c (%)	4.91±0.85	5.27±1.06	$7.17\pm1.26^{\#\#\blacktriangle\blacktriangle}$

EF (%)	64.25±2.26	62.20±5.28	58.55±5.67##▲

Supplementary Table 2. The basic baseline characteristics of non-diabetic, pre-diabetic and diabetic patients as follows. Values are expressed as Mean± SD. P values refer to Student t and χ2 tests. BMI, body mass index; SBP, Systolic blood pressure; DBP, diastolic blood pressure; TG, total triglyceride; TC, total cholesterol; HDL-C, high density lipoprotein cholesterol; LDL-C, low density lipoprotein cholesterol; FPG, fasting plasma glucose; HbA1c, glycated hemoglobin A1c; EF, ejection fraction. ** $p < 0.01$, Pre-DM vs. Non-DM; ## $p < 0.01$, DM vs. Non-DM; ▲ $p < 0.05$, ▲▲ $p < 0.01$, DM vs. Non-DM.

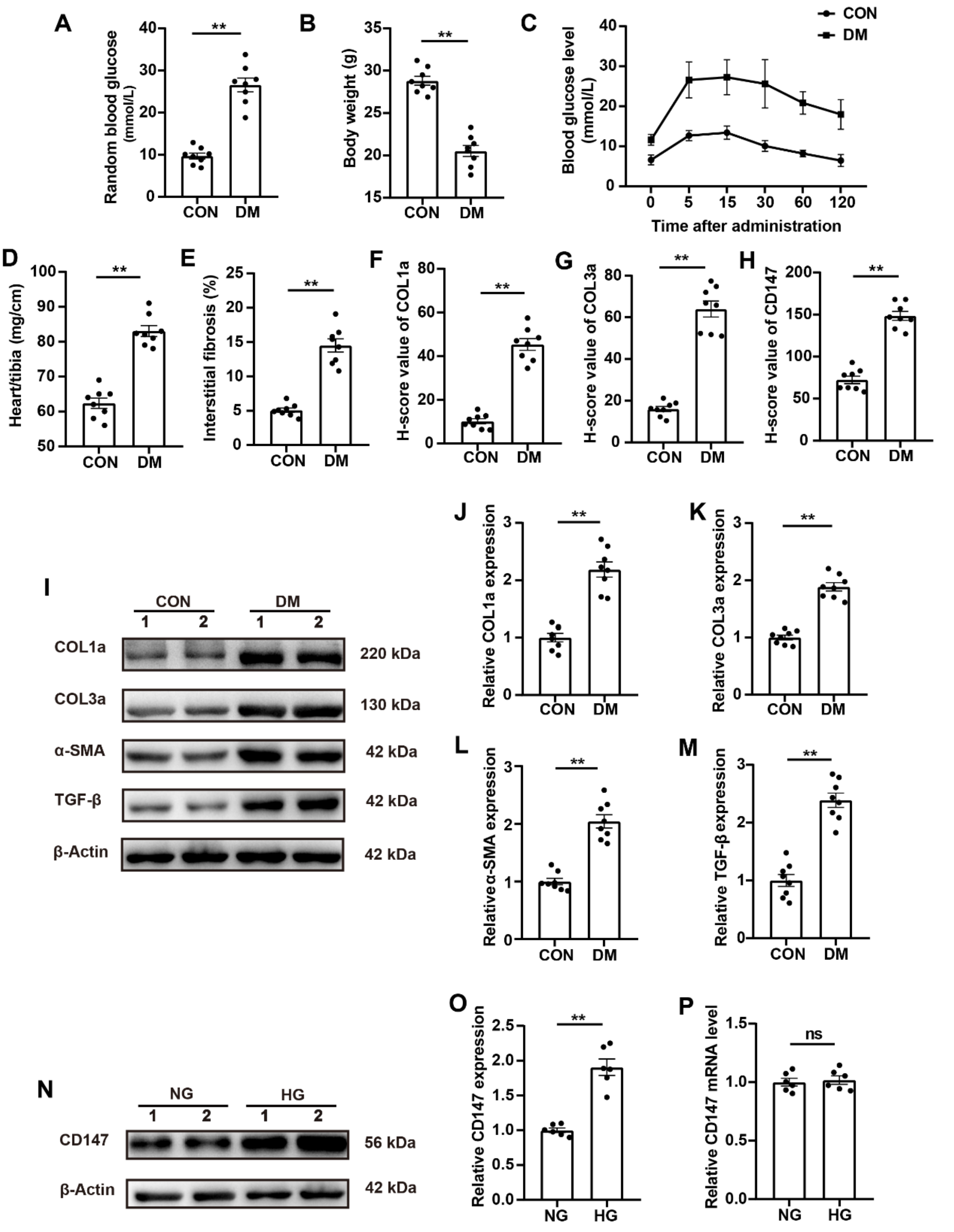

Supplementary Fig. 1. STZ-induced diabetic hearts exhibit excessive collagen deposition and upregulated CD147 expression. (A-C) Random blood glucose level, body weight, glucose

tolerance compared between control and diabetic mice. n = 8 mice. **(D)** The ratio of heart weight to tibia length. n = 8 mice. **(E)** The quantification of interstitial fibrosis area. n = 8 mice. **(F-H)** The quantification of immunohistochemical staining for COL1a, COL3a and CD147 in heart sample. n = 8 mice. **(I-M)** Representative western blot and analysis of COL1a, COL3a, α-SMA, TGF-β in mice hearts. n = 8 mice. **(N-P)** Representative western blot and real-time PCR analysis of CD147 in primary cardiac fibroblasts. n = 6 wells in each group. Data represent as mean ± SEM. *$p < 0.05$, **$p < 0.01$.

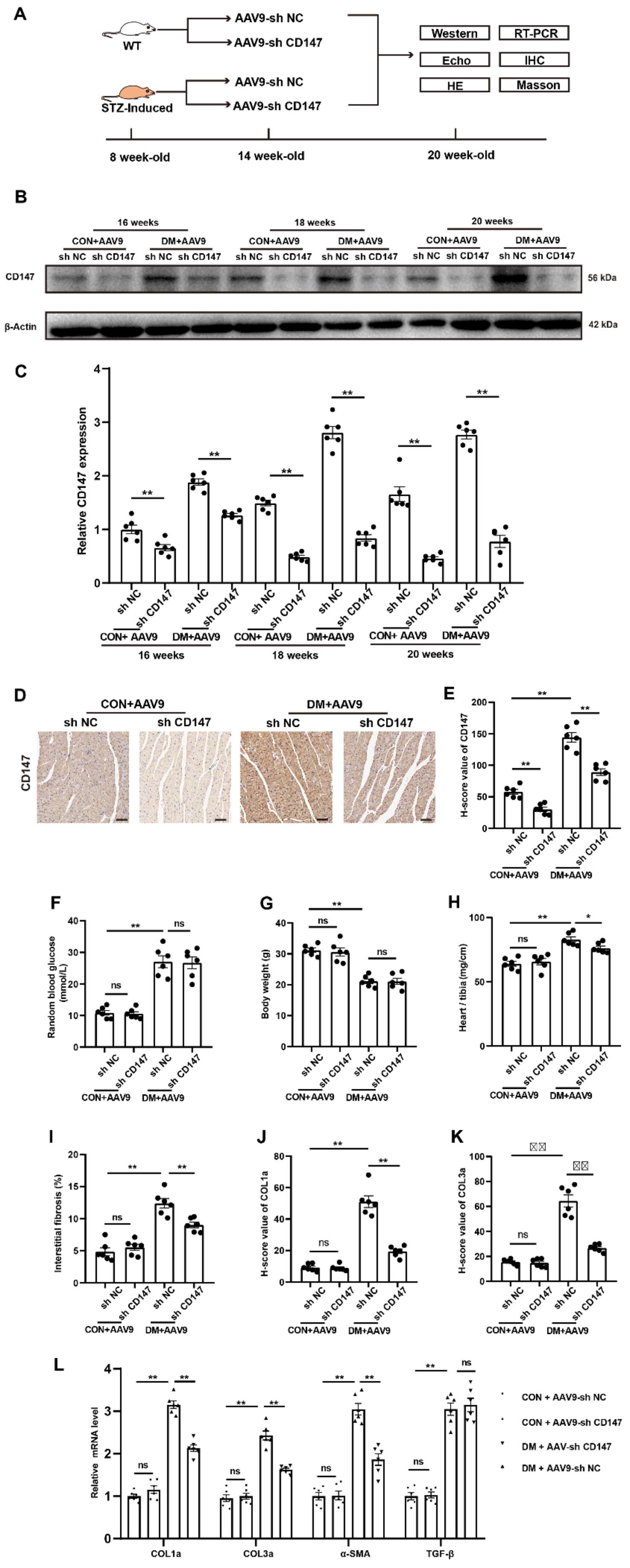
A
WT
STZ-Induced
AAV9-sh NC
AAV9-sh CD147
Western
RT-PCR
Echo
IHC
HE
Masson
8 week-old
14 week-old
20 week-old
B
16 weeks
18 weeks
20 weeks
CON+AAV9
DM+AAV9
sh NC
sh CD147
CD147
β-Actin
56 kDa
42 kDa
C
Relative CD147 expression
D
CD147
E
H-score value of CD147
F
Random blood glucose (mmol/L)
G
Body weight (g)
H
Heart / tibia (mg/cm)
I
Interstitial fibrosis (%)
J
H-score value of COL1a
K
H-score value of COL3a
L
Relative mRNA level
COL1a
COL3a
α-SMA
TGF-β
CON + AAV9-sh NC
CON + AAV9-sh CD147
DM + AAV-sh CD147
DM + AAV9-sh NC
ns
**
*

Supplementary Fig. 2. Silencing CD147 alleviates cardiac dysfunction and fibrosis in diabetic hearts. (A) Schematic diagram of the experimental process. **(B, C)** Representative western blot and analysis of CD147 expression in mice hearts at 16 weeks, 18 weeks and 20 weeks. **(D, E)** Representative immunohistochemical staining and analysis of CD147 in mice hearts at 20 weeks. Scale bar=30 μm. **(F, G)** Random blood glucose level, body weight of mice. **(H)** The ratio of heart weight to tibia length. **(I)** The quantification of interstitial fibrosis area. **(J, K)** The quantification of immunohistochemical staining for COL1a, COL3a and CD147 in heart sample. **(L)** Real-time PCR analysis of COL1a, COL3a, α-SMA, TGF-β in mice hearts. n = 6 mice in each group. Data represent as mean ± SEM. *p < 0.05, **p < 0.01.

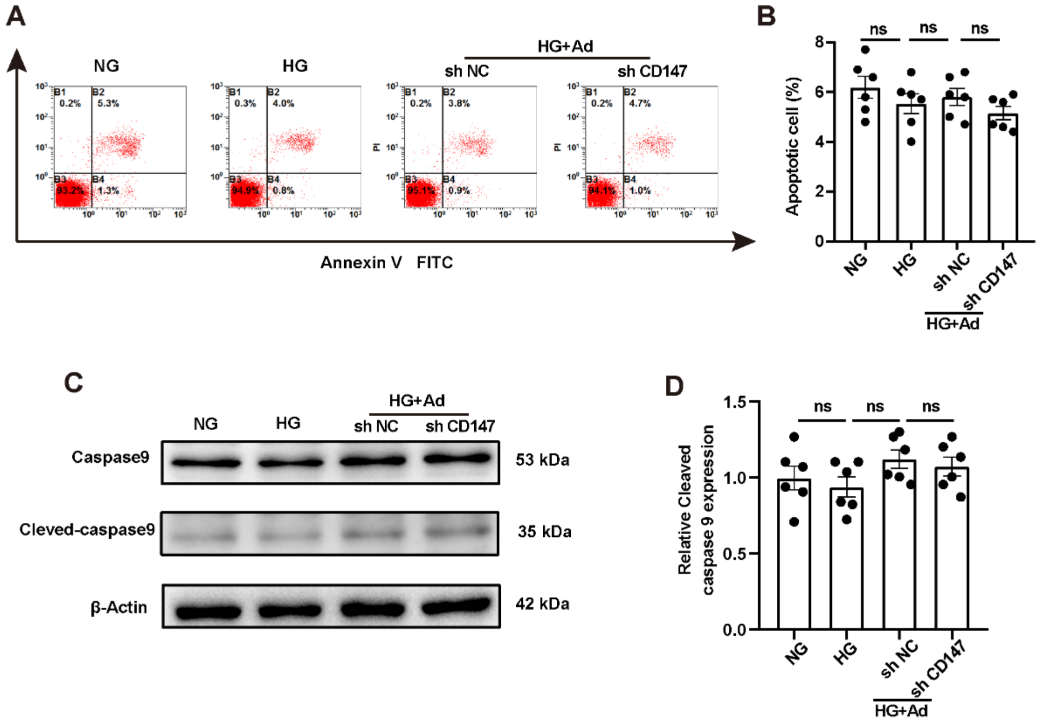

Supplementary Fig. 3. CD147 is involved in HG-induced activation of CFs. (A, B) Annexin V and PI staining for flow cytometry were used to determine the apoptosis cells of primary myocardial fibroblasts. **(C, D)** Representative western analysis of Cleaved caspase 9 in primary cardiac fibroblasts. n = 6 wells in each group. Data represent as mean ± SEM. *p < 0.05, **p < 0.01.

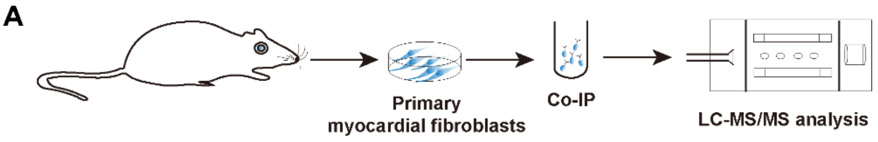

Protein name	Unique peptides	Molecular Mass (KDa)	Abundance	Ms score
ALK5	33	55	2.5×10^8	88
Atg2	24	42	1.3×10^7	67
Myh10	18	220	1.1×10^7	53
MCT4	15	45	3.3×10^6	51
Krt13	9	49	2.2×10^6	43

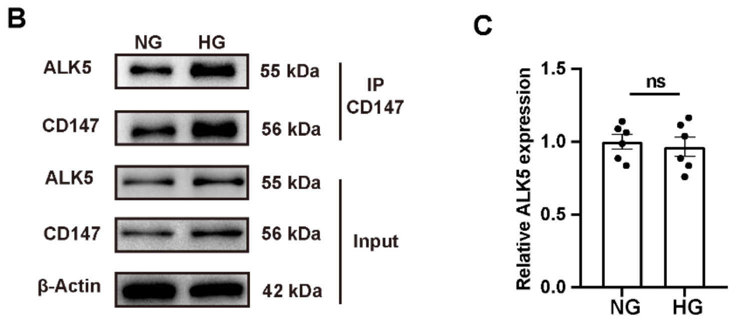

Supplementary Fig. 4. CD147 facilitates ALK5 activation and endocytosis to regulate HG-induced SMAD2/3 phosphorylation and nuclear translocation. (A) Schematic diagram of the experimental process. **(B)** Mass spectrometry analysis of CD147 interacting proteins. Atg2, autophagy-related protein 2; Myh10, myosin-10; MCT4, monocarboxylate transporter 4; Krt13, type I cytoskeletal 13. **(C, D)** The binding capacity of CD147 to ALK5 was demonstrated by Co-IP. n = 6 wells in each group. Data represent as mean ± SEM. *p < 0.05, **p < 0.01.

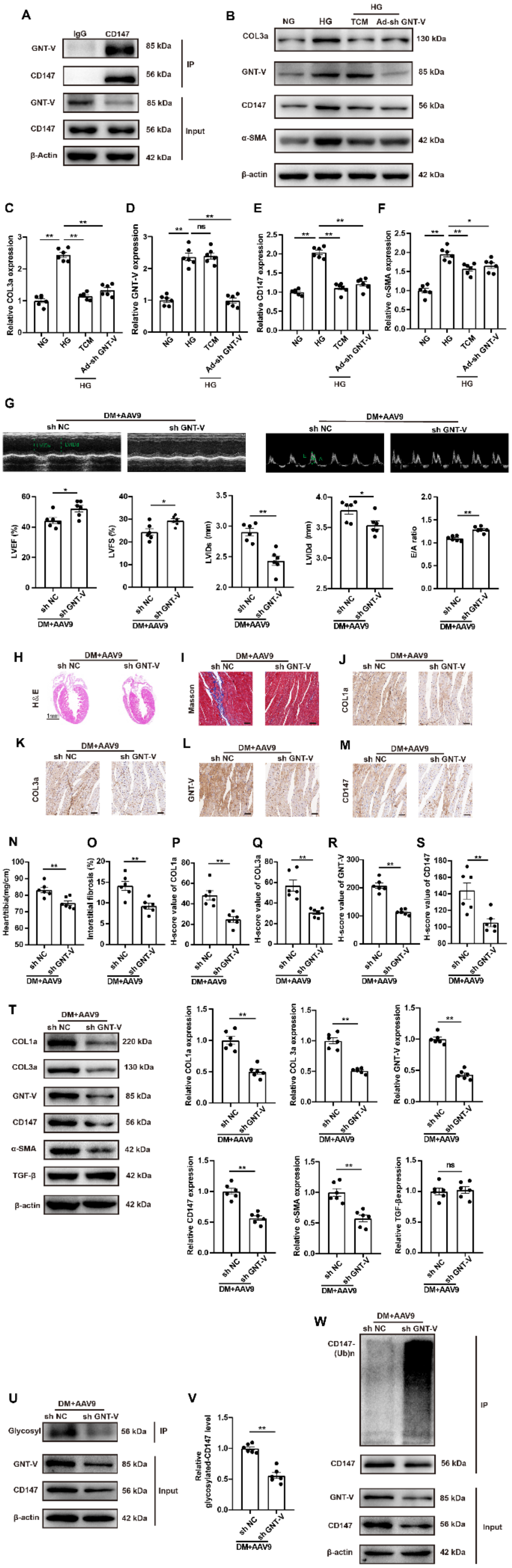

A
IgG
CD147
GNT-V
CD147
GNT-V
CD147
β-Actin
85 kDa
56 kDa
85 kDa
56 kDa
42 kDa
IP
Input
B
HG
NG
HG
TCM
Ad-sh GNT-V
COL3a
GNT-V
CD147
α-SMA
β-actin
130 kDa
85 kDa
56 kDa
42 kDa
42 kDa
C
Relative COL3a expression
D
Relative GNT-V expression
E
Relative CD147 expression
F
Relative α-SMA expression
NG
HG
TCM
Ad-sh GNT-V
HG
G
DM+AAV9
sh NC
sh GNT-V
LVEF (%)
LVFS (%)
LVIDs (mm)
LVIDd (mm)
E/A ratio
H
H&E
I
Masson
J
COL1a
K
COL3a
L
GNT-V
M
CD147
N
Heart/tibia(mg/cm)
O
Interstitial fibrosis (%)
P
H-score value of COL1a
Q
H-score value of COL3a
R
H-score value of GNT-V
S
H-score value of CD147
T
COL1a
COL3a
GNT-V
CD147
α-SMA
TGF-β
β-actin
220 kDa
130 kDa
85 kDa
56 kDa
42 kDa
42 kDa
42 kDa
Relative COL1a expression
Relative COL 3a expression
Relative GNT-V expression
Relative CD147 expression
Relative α-SMA expression
Relative TGF-β expression
U
Glycosyl
GNT-V
CD147
β-actin
56 kDa
85 kDa
56 kDa
42 kDa
IP
Input
V
Relative glycosylated-CD147 level
W
CD147-(Ub)n
CD147
GNT-V
CD147
β-actin
56 kDa
85 kDa
56 kDa
42 kDa
IP
Input

Supplementary Fig. 5. HG promotes CD147 glycosylation and suppresses its ubiquitin-proteasomal degradation. (A) Interaction between CD147 and GNT-V was demonstrated by Co-IP. **(B-F)** Representative western analysis of COL3a, GNT-V, CD147, α-SMA in primary cardiac fibroblasts. n = 6 wells in each group. **(G)** Representative images and analysis of echocardiography; LVIDs, LVIDd and E/A ratio are marked (green). **(H)** Representative H&E staining of hearts. Scale bar=1 mm; **(I)** Representative Masson's trichrome staining of hearts. Scale bar=30 μm. **(J-M)** Representative immunohistochemical staining of COL1a, COL3a, GNT-V and CD147 in mice hearts. Scale bar=30 μm. **(N)** The ratio of heart weight to tibia length. **(O)** The quantification of interstitial fibrosis area. **(P-S)** The quantification of immunohistochemical staining for COL1a, COL3a, GNT-V and CD147 in heart sample. **(T)** Representative western blot and analysis of COL1a, COL3a, GNT-V, CD147, α-SMA and TGF-β in mice hearts. **(U-V)** Glycosylation level of CD147 were determined in mice hearts after GNT-V knock down. **(W)** IP assay and western blot analysis determined CD147 protein and ubiquitylation level in mice hearts after GNT-V knock down. n = 6 mice in each group. Data represent as mean ± SEM. $*p < 0.05$, $**p < 0.01$.

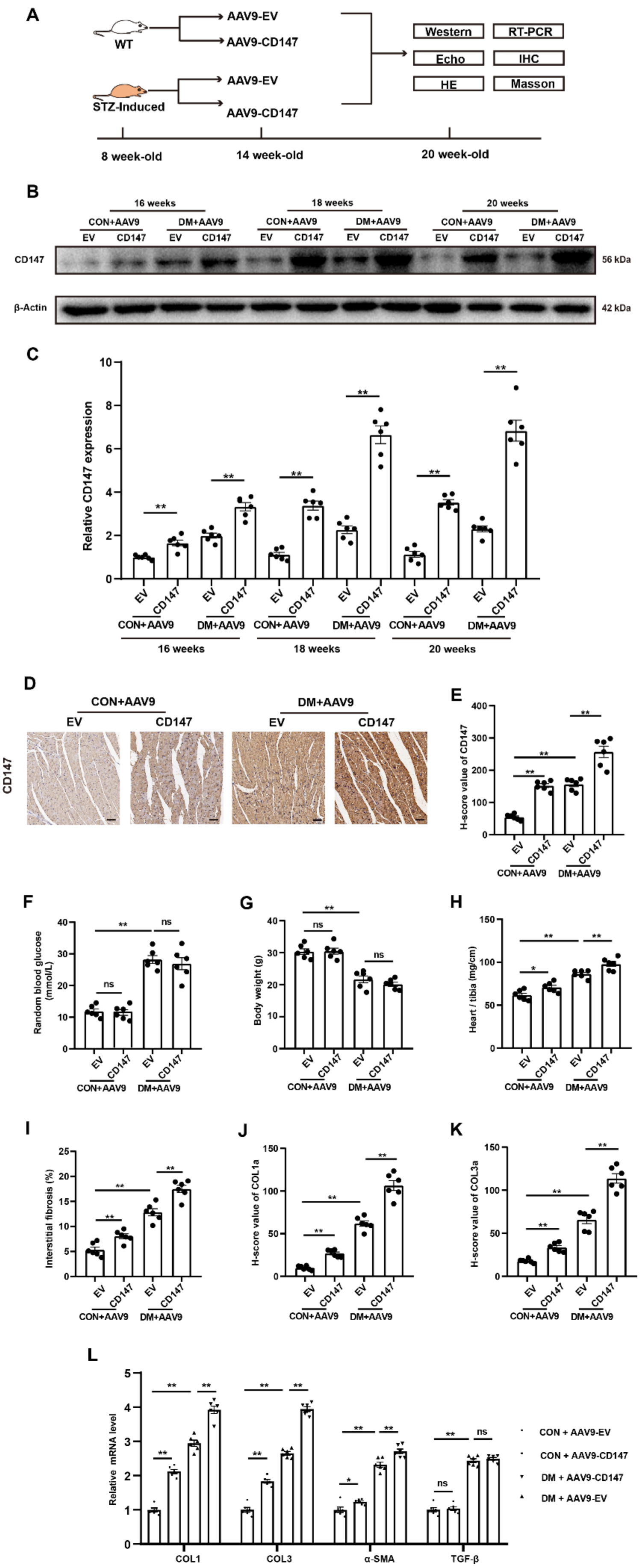
A
WT
STZ-Induced
AAV9-EV
AAV9-CD147
AAV9-EV
AAV9-CD147
Western
RT-PCR
Echo
IHC
HE
Masson
8 week-old
14 week-old
20 week-old
B
16 weeks
18 weeks
20 weeks
CON+AAV9
DM+AAV9
EV
CD147
CD147
β-Actin
56 kDa
42 kDa
C
Relative CD147 expression
D
CON+AAV9
DM+AAV9
E
H-score value of CD147
F
Random blood glucose (mmol/L)
G
Body weight (g)
H
Heart / tibia (mg/cm)
I
Interstitial fibrosis (%)
J
H-score value of COL1a
K
H-score value of COL3a
L
Relative mRNA level
COL1
COL3
α-SMA
TGF-β
CON + AAV9-EV
CON + AAV9-CD147
DM + AAV9-CD147
DM + AAV9-EV

Supplementary Fig. 6. CD147 overexpression in control mice mimicked diabetes-induced cardiac fibrosis. **(A)** Schematic diagram of the experimental process. **(B, C)** Representative western blot and analysis of CD147 expression in mice hearts at 16 weeks, 18 weeks and 20 weeks. **(D, E)** Representative immunohistochemical staining and analysis of CD147 in mice hearts at 20 weeks. Scale bar=30 μm. **(F, G)** Random blood glucose level, body weight of mice. **(H)** The ratio of heart weight to tibia length. **(I)** The quantification of interstitial fibrosis area. **(J, K)** The quantification of immunohistochemical staining for COL1a, COL3a and CD147 in heart sample. **(L)** Real-time PCR analysis of COL1a, COL3a, α-SMA, TGF-β in mice hearts. n = 6 mice in each group. Data represent as mean ± SEM. $^{*}p < 0.05$, $^{**}p < 0.01$.

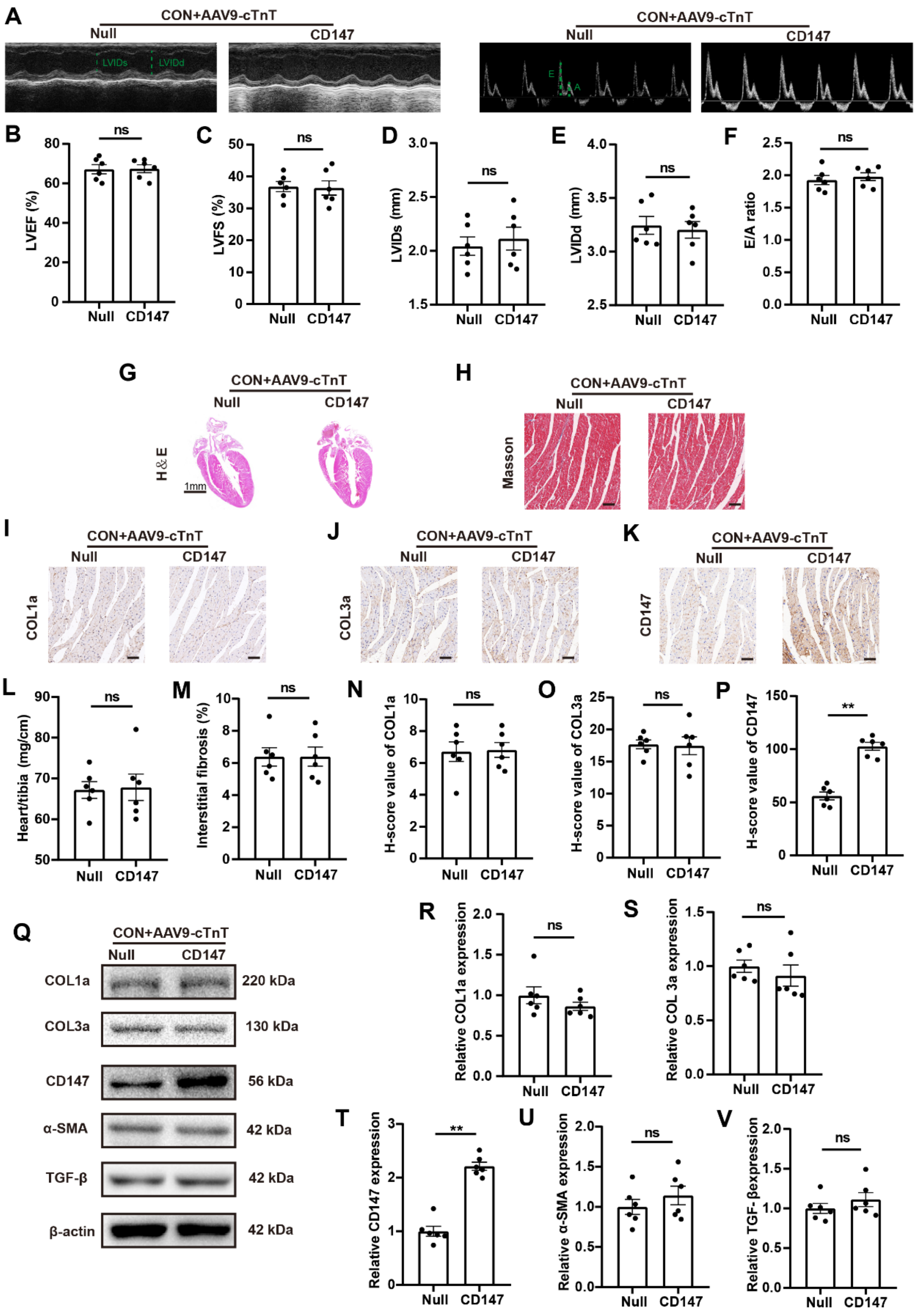

Supplementary Fig. 7. Targeting cardiomyocytes to overexpress CD147 in control mice failed to mimic cardiac dysfunction and fibrosis. (A-F) Representative images and analysis of echocardiography; LVIDs, LVIDd and E/A ratio are marked (green). **(G)** Representative H&E staining of hearts. Scale bar=1 mm; **(H)** Representative Masson's trichrome staining of hearts. Scale bar=30 μm. **(I-K)** Representative immunohistochemical staining of COL1a, COL3a and

CD147 in mice hearts. Scale bar=30 μm. **(L)** The ratio of heart weight to tibia length. **(M)** The quantification of interstitial fibrosis area. **(N-P)** The quantification of immunohistochemical staining for COL1a, COL3a and CD147 in heart sample. **(Q-V)** Representative western blot and analysis of COL1a, COL3a, CD147, α-SMA and TGF-β in mice hearts. n = 6 mice in each group. Data represent as mean ± SEM. **$p < 0.01$.
